# Autism-Related Transcription Factors Underlying the Sex-Specific Effects of Prenatal Bisphenol A Exposure on Transcriptome-Interactome Profiles in the Offspring Prefrontal Cortex

**DOI:** 10.3390/ijms222413201

**Published:** 2021-12-08

**Authors:** Songphon Kanlayaprasit, Surangrat Thongkorn, Pawinee Panjabud, Depicha Jindatip, Valerie W. Hu, Takako Kikkawa, Noriko Osumi, Tewarit Sarachana

**Affiliations:** 1 The Ph.D. Program in Clinical Biochemistry and Molecular Medicine, Department of Clinical Chemistry, Faculty of Allied Health Sciences, Chulalongkorn University, Bangkok 10330, Thailand; songphon.ka@student.chula.ac.th (S.K.); 6176957037@student.chula.ac.th (S.T.); 6176952837@student.chula.ac.th (P.P.); 2 Systems Neuroscience of Autism and PSychiatric Disorders (SYNAPS) Research Unit, Department of Clinical Chemistry, Faculty of Allied Health Sciences, Chulalongkorn University, Bangkok 10330, Thailand; depicha.j@chula.ac.th; 3 Department of Anatomy, Faculty of Medicine, Chulalongkorn University, Bangkok 10330, Thailand; 4 Department of Biochemistry and Molecular Medicine, School of Medicine and Health Sciences, The George Washington University, Washington, DC 20052, USA; valhu@gwu.edu; 5 Department of Developmental Neuroscience, United Centers for Advanced Research and Translational Medicine (ART), Tohoku University Graduate School of Medicine, Sendai 980-8577, Miyagi, Japan; kikkawa@med.tohoku.ac.jp (T.K.); osumi@med.tohoku.ac.jp (N.O.)

**Keywords:** endocrine-disrupting chemical, bisphenol A, prenatal exposure, autism spectrum disorder, sex differences, transcription factor, transcriptome, interactome, prefrontal cortex, molecular docking

## Abstract

Bisphenol A (BPA) is an environmental risk factor for autism spectrum disorder (ASD). BPA exposure dysregulates ASD-related genes in the hippocampus and neurological functions of offspring. However, whether prenatal BPA exposure has an impact on genes in the prefrontal cortex, another brain region highly implicated in ASD, and through what mechanisms have not been investigated. Here, we demonstrated that prenatal BPA exposure disrupts the transcriptome–interactome profiles of the prefrontal cortex of neonatal rats. Interestingly, the list of BPA-responsive genes was significantly enriched with known ASD candidate genes, as well as genes that were dysregulated in the postmortem brain tissues of ASD cases from multiple independent studies. Moreover, several differentially expressed genes in the offspring’s prefrontal cortex were the targets of ASD-related transcription factors, including AR, ESR1, and RORA. The hypergeometric distribution analysis revealed that BPA may regulate the expression of such genes through these transcription factors in a sex-dependent manner. The molecular docking analysis of BPA and ASD-related transcription factors revealed novel potential targets of BPA, including RORA, SOX5, TCF4, and YY1. Our findings indicated that prenatal BPA exposure disrupts ASD-related genes in the offspring’s prefrontal cortex and may increase the risk of ASD through sex-dependent molecular mechanisms, which should be investigated further.

## 1. Introduction

Autism spectrum disorder (ASD) is a neurodevelopmental disorder that can be diagnosed in early childhood and is characterized by two behavioral domains, i.e., social communication/interaction deficits and restricted interests or repetitive patterns of behavior according to the Diagnostic and Statistical Manual of Mental Disorders, 5th Edition (DSM-5) [1]. In the United States, the Centers for Disease Control and Prevention (CDC) estimated the prevalence of ASD in children by the age of eight to be 1 in 54, with at least four times higher in males than in females [2]. Although the exact causes and the sex bias of ASD are still unclear, there is accumulating evidence that genetic, epigenetic, and environmental factors are involved in ASD etiology and susceptibility [3,4,5,6,7,8]. Environmental factors that are associated with ASD include endocrine-disrupting chemicals (EDCs) [5,9,10], heavy metals [11,12], smoke from cigarettes [13], and traffic-related air pollutants [14]. EDCs are chemical substances that potentially affect the hormonal system and disrupt homeostasis, reproduction, and developmental processes [5]. Examples of EDCs that have been linked to ASD are bisphenol A (BPA) [15,16], polychlorinated biphenyls (PCBs) [17], phthalates [18], and polybrominated diphenyl ethers (PBDEs) [19]. BPA ((CH_3_)_2_C(C_6_H_4_OH)_2_) is an organic synthetic compound frequently found in polycarbonate plastic and epoxy resin products, the linings inside beverages and food cans, thermal paper, and dental sealants. In addition to such products, BPA is also found in polycarbonate micro/nanoplastics, which have become one of the major environmental problems worldwide [20,21]. When these products or micro/nanoplastics are exposed to high temperature or basic/acidic conditions, BPA can leach out and contaminate food or pollute the environment [20,22,23,24]. When ingested, the majority of BPA is converted to a conjugated form called BPA glucuronide in the liver and is then excreted through the urinary system [25,26]. The unconjugated BPA molecules remain circulating in the bloodstream and other tissues in the body [27,28,29]. Recent studies have shown that BPA can cross the placenta [30,31] and the blood–brain barriers [32,33] to reach the brains of offspring [34]. Recent findings have shown that humans are widely exposed to BPA [35,36]. The National Health and Nutrition Examination Survey (NHANES III) conducted by the CDC in 2003–2004 found detectable levels of BPA in as many as 93% of 2517 urine samples from participants who were at the age of six years and older [37]. Similar to a previous study conducted by the CDC, Hansen et al. found that BPA was detected in as many as 85.3% of maternal urine samples [38]. In addition to urine, other studies reported that BPA is also detectable in maternal and fetal sera [39], amniotic fluid [40], placenta [41], umbilical cords [42], colostrum [43], and breast milk [44]. In ASD populations, elevated levels of BPA have been observed in both the blood and urine of ASD cases [45,46,47]. Kardas et al. measured BPA concentrations in the serum of ASD and typically developing children using high-performance liquid chromatography (HPLC) and found that children with ASD have significantly higher serum BPA concentrations than typically developing children [46]. Similarly, Kondolot et al. found elevated levels of BPA in the plasma of children with pervasive developmental disorder not otherwise specified (PDD-NOS), which is a subtype of ASD [45]. Hansen et al. assessed the association between in utero exposure to BPA and ASD symptoms in Danish children and observed elevated odds ratios among 5-year-old children within the 3rd tertile of BPA exposure with an ASD score above the 75th percentile [38]. Their finding supports the hypothesis that prenatal BPA exposure may increase the risk of ASD symptoms. However, the effects of prenatal BPA exposure and the underlying molecular mechanisms in the context of ASD remain unclear.

The U.S. Food and Drug Administration (FDA) has determined the no-observed-adverse-effect level (NOAEL) of BPA to be 5000 µg/kg maternal body weight/day [48,49]. However, there is accumulating evidence that exposure to BPA, even at the NOAEL dose or lower, can cause negative health effects [50,51,52,53]. Several studies have reported that BPA exposure has negative impacts on the central nervous system [54,55], the reproductive system [56], the immune system [57], the digestive system [58], the thyroid [59], the liver [60], the heart [61], kidneys [62], and adipose tissues [63]. In the central nervous system, BPA alters neuronal viability [16], neuronal morphology [64], synaptogenesis [65], synaptic densities [65], and neocortical development [66,67]. It also causes behavioral alterations, including reduced social interaction [68], repetitive behaviors [69], impaired learning/memory [16], increased anxiety [70], hyperactivity [71], and inattention [72], all of which are associated with ASD [73,74,75,76]. However, the molecular mechanisms underlying the effects of BPA, particularly the neurological functions and behaviors associated with ASD, are still unclear.

Recent studies have shown that BPA exposure disrupts the expression of genes in several brain regions, including the prefrontal cortex [77], the hippocampus [15,78], the hypothalamus [78], the cerebellum [79], and the amygdala [80]. The prefrontal cortex is a brain region responsible for executive functions, including decision making, planning, prediction of outcomes, and social behavior, all of which are known to be impaired in people with ASD [81,82,83]. Moreover, several studies have shown that children with ASD exhibit an excess number of neurons [84] and decreased functional connectivity in the prefrontal cortex [85]. Using adult rats as a model, Castro et al. examined the effects of adult exposure to BPA on the expression of genes and proteins in the prefrontal cortex by real-time (RT)-PCR and Western blot analyses [77]. They found that BPA exposure increases the expression of cytochrome P450 aromatase and tryptophan hydroxylase 2 in male and female rats and decreases the expression of 5α-reductase in females [77]. In addition to these genes, they also found that a total of 17 genes associated with prefrontal cortex functions are responsive to adult BPA exposure, indicating that BPA can alter the expression profiles of multiple genes in the prefrontal cortex region. It is noteworthy that cytochrome P450 aromatase—an enzyme responsible for estrogen biosynthesis from androgens—is associated with ASD and thought to be involved in the male bias of the disorder [86,87,88]. However, this study was focused on adult exposure and on genes known to be involved in drug- and chemical-induced neurotoxic responses. The effects of BPA on the transcriptome profiles of the prefrontal cortex and its association with ASD are unclear.

Our recent study demonstrated that prenatal BPA exposure alters the transcriptome–interactome profiles of genes in the hippocampus of rat offspring [15]. Among those BPA-responsive genes are genes known to be associated with ASD and related neurological functions [15]. In addition, we also found that prenatal BPA exposure disrupts ASD-related genes involved in neuronal viability, neuritogenesis, and learning/memory in a sex-dependent manner [16], suggesting that prenatal BPA exposure may play an important role in the risk and sex difference of ASD. However, the effects and mechanisms of prenatal BPA exposure on the transcriptome–interactome profiles of ASD-related genes in the prefrontal cortex have not been investigated.

In this study, we therefore sought to interrogate the effects and mechanisms of prenatal BPA exposure on the transcriptome–interactome profiles of genes in the prefrontal cortex of offspring, as well as determining the association of BPA-responsive genes and ASD. First, the RNA sequencing (RNA-seq) analysis of prefrontal cortex tissues isolated from neonatal rats prenatally exposed to BPA or vehicle control was performed. Then, the lists of differentially expressed genes (DEGs) from the RNA-seq analysis were analyzed by Ingenuity Pathway Analysis (IPA) software (QIAGEN Inc., Hilden, Germany, https://www.qiagenbioinformatics.com/products/ingenuity-pathway-analysis/, accessed on 14 June 2019) [89] to predict biological functions and interactome networks associated with BPA-responsive genes and to determine whether BPA-responsive genes are associated with ASD. The link between the DEGs in the prefrontal cortex and ASD was also assessed by hypergeometric distribution analyses with the lists of ASD candidate genes from the ASD database SFARI. To further investigate whether these BPA-responsive genes were also found to be disrupted in the brains of ASD patients, we conducted a meta-analysis using transcriptome profiles of postmortem brain tissues from ASD patients and typically developing people from multiple independent studies that were published in the NCBI GEO Dataset database. The lists of BPA-responsive genes were then compared to DEGs in brain tissues of ASD cases using hypergeometric distribution analyses. BPA-responsive genes associated with ASD were selected for qRT-PCR analysis. To predict transcription factors involved in the effects of BPA on the transcriptome profiles in the prefrontal cortex, a list of transcription factors and the targets of each transcription factor were obtained and used for prediction. The molecular docking analysis of BPA and ASD-related transcription factors of which the targets were significantly enriched in the lists of BPA-responsive genes was further conducted to predict the binding affinity.

## 2. Results

### 2.1. Prenatal BPA Exposure Disrupts the Transcriptome Profiles of the Offspring’s Prefrontal Cortex in a Sex-Dependent Manner

To investigate whether prenatal BPA exposure alters the transcriptome profiles of the offspring’s prefrontal cortex, rat dams were treated with 5000 µg/kg maternal body weight or vehicle control daily using oral gavage from gestational day (GD) 1 until parturition. The dose of BPA used to treat rat dams in this study was equal to the NOAEL in humans as determined by the FDA. RNA-seq analysis was then performed using prefrontal cortex tissues isolated from neonatal male and female pups. To identify significantly differentially expressed transcripts in response to BPA, the RNA-seq data of male and female pups were then analyzed by combining both sexes into one group for each treatment (Table S1). The data of each sex of pups were also analyzed separately to gain insight into the sex difference in the effects of prenatal BPA exposure (Tables S2 and S3).

When the RNA-seq data of male and female pups in each treatment group were combined, a total of 16,182 transcripts corresponding to 14,144 genes were detectable in the prefrontal cortex of the pups. Among these, 7810 transcripts encoding 6284 genes were more significantly differentially expressed in the prefrontal cortex of rats prenatally exposed to BPA compared with in that of the controls (*p*-value < 0.05 and false discovery rate (FDR) < 0.05). When each sex was analyzed separately, a total of 16,782 transcripts encoding 14,672 genes and 16,819 transcripts encoding 14,660 genes were detectable in the prefrontal cortex of male and female pups, respectively. We found that 3728 transcripts (corresponding to 2565 genes) were significantly differentially expressed in males, whereas 3830 transcripts (corresponding to 2706 genes) were differentially expressed in females in response to BPA (*p*-value < 0.05 and FDR < 0.05). The lists of DEGs are shown in Tables S1–S3. This finding indicates that prenatal BPA exposure alters the transcriptome profiles of offspring’s prefrontal cortex in a sex-dependent manner.

### 2.2. BPA-Responsive Genes in the Prefrontal Cortex Are Associated with ASD and Related Neurological Functions and Pathways

To determine whether genes differentially expressed in the offspring’s prefrontal cortex in response to prenatal BPA exposure are associated with ASD or related biological functions and pathways, gene ontology analysis was performed using IPA software (Table 1, Tables S4 and S5).

Interestingly, we found that genes associated with “autism or intellectual disability” were significantly enriched in the lists of DEGs in the combined group (233 genes; *p*-value = 4.11 × 10^−11^), male-only group (128 genes; *p*-value = 1.89 × 10^−13^), and female-only group (105 genes; *p*-value = 2.31 × 10^−5^) (Table 1). In addition, DEGs were also associated with other neurological disorders known to be comorbid disorders of ASD, including pervasive developmental disorder, schizophrenia spectrum disorder, movement disorders, and syndromic encephalopathy (*p*-value < 0.05). The biological function and canonical pathway analysis by IPA revealed that DEGs were related to neurological functions and pathways known to be disrupted in ASD, including axonal guidance signaling, PTEN signaling, synaptic long-term depression and potentiation, and Wnt/calcium signaling (*p*-value < 0.05; Tables S4 and S5). “Proliferation of neuronal cells”, “neuritogenesis”, and “neurotransmission” were also highlighted. Notably, BPA-responsive genes in the prefrontal cortex of male pups, but not female pups, were also significantly associated with androgen signaling (63 genes; *p*-value = 2.95 × 10^−4^) and estrogen receptor signaling (50 genes; *p*-value = 0.02) (Table S5), both of which have been implicated in the male bias of ASD.

To visualize the interactions among DEGs in the prefrontal cortex as well as the association between DEGs and diseases/functions associated with ASD, an interactome network analysis was performed using IPA software (Figure S1). The interactome network of DEGs from the both-sex group showed the interaction of DEGs and ASD-related pathological conditions, including “intellectual disability” and “sensory and motor neuropathy”. Notably, *Lnpk*—a gene associated with autistic features—was shown to be the hub gene of the interactome network (Figure S1A). The interactome networks of DEGs in male and female pups prenatally exposed to BPA also showed associations with neurological disorders and functions linked to the development of the prefrontal cortex and ASD, including “autism spectrum disorder”, “neurodevelopmental disorder”, “developmental delay”, “learning”, “formation of the forebrain”, “development of cortical plate”, and “neuritogenesis” (Figure S1B,C). These findings suggest that prenatal exposure to BPA alters the expression profiles of genes associated with ASD or related neurological functions in the prefrontal cortex in a sex-specific pattern.

### 2.3. Known ASD Candidate Genes Are Significantly Enriched in the Lists of BPA-Responsive Genes in the Prefrontal Cortex

To further examine whether BPA-responsive genes are significantly associated with genes previously reported in the literature to be ASD candidate genes, the lists of DEGs in the offspring’s prefrontal cortex were compared to the lists of known ASD candidate genes obtained from the ASD database SFARI. Hypergeometric distribution analysis was then performed to determine the statistical significance of the association between DEGs and known ASD candidate genes with different confidence levels determined by the SFARI database (Table 2).

Consistent with the gene ontology analysis using IPA, known ASD candidate genes with high confidence levels identified by the SFARI database as “syndromic”, “high confidence”, and “strong candidate” genes were significantly enriched in the lists of DEGs in the prefrontal cortex of male and female pups prenatally exposed to BPA. When both sexes of pups were combined into one group for each treatment, as many as 408 known ASD candidate genes were significantly enriched in the list of DEGs (*p*-value = 4.44 × 10^−3^). The highly significant associations between the lists of DEGs and the known ASD candidate genes were also observed, when male and female rat pups were analyzed separately (243 genes, *p*-value = 6.64 × 10^−17^ in males; 228 genes, *p*-value = 1.70 × 10^−10^ in females). The lists of DEGs that are the known ASD candidate genes as well as the confidence categories identified by the SFARI database are shown in Tables S6–S8.

**Figure 1:**
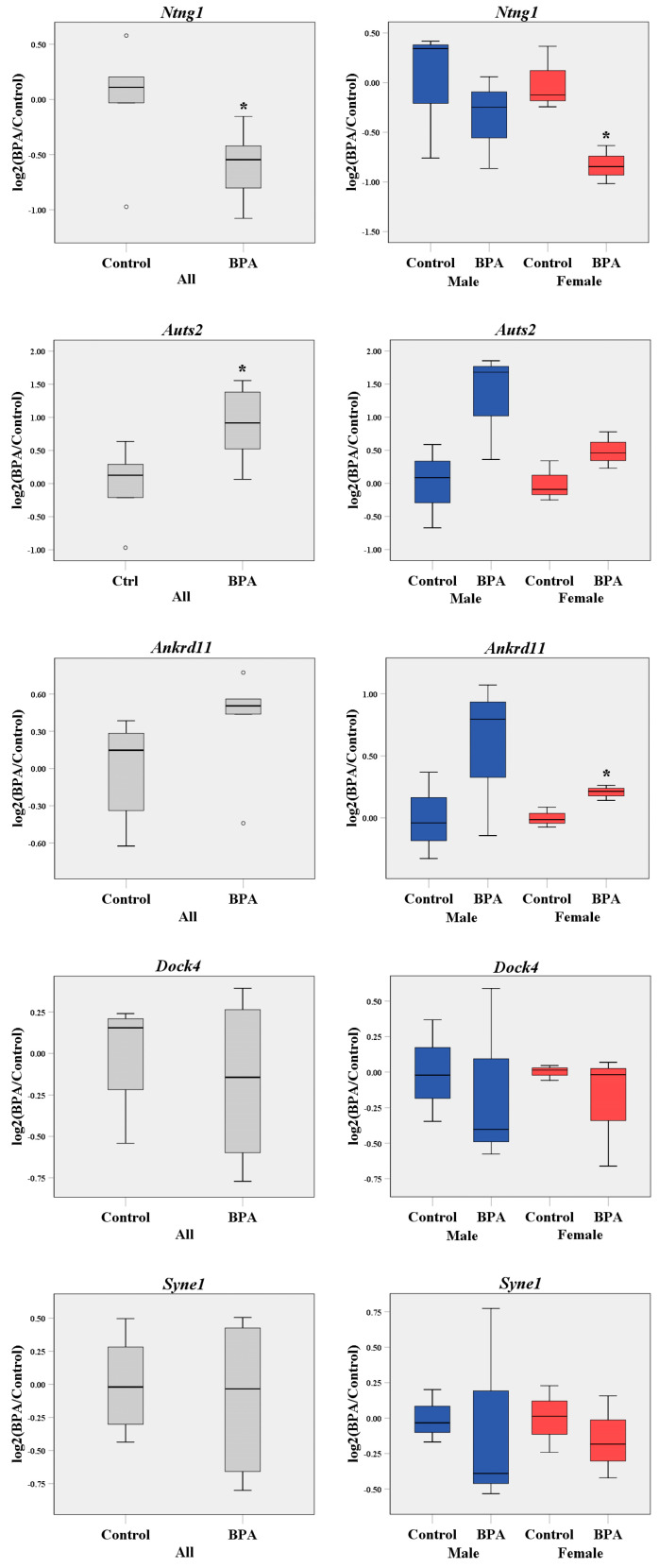
Box plot of autism spectrum disorder (ASD)-related gene expression in the prefrontal cortex tissues. The expression levels of *Ntng1*, *Auts2*, *Ankrd11*, *Dock4*, and *Syne1* were determined in both sexes (*n* = 6 pups/group; male pups = 3 and female pups = 3) and separately in males and females (*n* = 3 pups/sex/treatment group). * *p*-value < 0.05.

To further confirm that prenatal BPA exposure dysregulates the expression of ASD-related genes in the offspring’s prefrontal cortex, five ASD candidate genes, which were *Ntng1* (netrin G1), *Auts2* (autism susceptibility gene 2 or activator of transcription and developmental regulator AUTS2), *Ankrd11* (ankyrin repeat domain 11), *Dock4* (dedicator of cytokinesis 4), and *Syne1* (spectrin repeat containing nuclear envelope protein 1), were selected for qRT-PCR analysis (Figure 1). These ASD candidate genes were selected, because they are known to be abundantly expressed in the cortex during embryogenesis and involved in neuronal development during the prenatal period [90,91,92,93,94,95]. We found that *Ntng1* was significantly reduced whereas *Auts2* and *Ankrd11* were upregulated in response to prenatal BPA exposure (Figure 1). Interestingly, the significant downregulation of *Ntng1* and the upregulation of *Ankrd11* were observed in female pups only.

### 2.4. BPA-Responsive Genes Are Significantly Associated with DEGs in Postmortem Brain Tissues from ASD Cases

Next, we sought to further investigate whether DEGs in response to prenatal BPA exposure in the offspring’s prefrontal cortex were also found to be dysregulated in the brains of ASD patients. The transcriptome profiling data of postmortem brain tissues from people with ASD and typically developing people were obtained from the NCBI GEO database and reanalyzed using MeV software to identify genes that were significantly differentially expressed in ASD brain tissues. The details of transcriptome datasets and human brain samples are provided in Table S9. The lists of BPA-responsive genes in the rat offspring prefrontal cortex were then compared with DEGs in ASD brain tissues, and hypergeometric distribution analyses were performed to assess the statistical significance of the association. Interestingly, we found that BPA-responsive genes were significantly associated with DEGs in ASD brain samples from multiple independent transcriptomic profiling datasets, particularly with DEGs in ASD frontal and temporal cortex tissues (Table 3). The lists of BPA-responsive genes in the rat offspring prefrontal cortex that were also found to be dysregulated in ASD brain tissues from each transcriptomic dataset are shown in Tables S10–S12.

### 2.5. Identification of Upstream Regulators of DEGs in Response to Prenatal BPA Exposure

Recent studies have shown that BPA can interact with several transcription factors, including AR [96], ESR1 [97], ERRG [97], PPARG [97], and THRA [98]. However, it is still unclear what transcription factors are responsible for the neurotoxicity mediated by prenatal BPA exposure. To identify potential transcription factors through which BPA exerts its negative effects on transcriptome profiles of the offspring’s prefrontal cortex, the list of all known transcription factors was obtained from the Human Transcription Factors database [99]. Out of 1639 transcription factors available in the Human Transcription Factors database, a total of 96 transcription factors are associated with ASD and found in the SFARI database [100]. Then, the targets of each transcription factor were obtained from the TRANSFAC Curated, TRANSFAC Predicted, CHEA, ENCODE, JASPAR Predicted, and MotifMap Predicted databases through the Harmonizome database (Table S13). Among 96 ASD-related transcription factors, the targets of 34 transcription factors are available in the Harmonizome database, with 14 transcription factors of which the target genes are manually curated by the TRANSFAC Curated Transcription Factor Targets database [101,102]. We then performed the hypergeometric distribution analysis between the lists of BPA-responsive genes and the targets of each transcription factor available in the Harmonizome database to examine whether the targets of ASD-related transcription factors were significantly enriched in the lists of BPA-responsive genes. The hypergeometric distribution analysis revealed the significant associations between BPA-responsive genes and the targets of 25 transcription factors (Table S13). Out of 25 significant transcription factors, the targets of 10 transcription factors, including AR, ESR1, RORA, SOX5, TCF4, and YY1, are manually curated and available in the TRANSFAC Curated database (Table 4).

To further examine whether the targets of RORA in neuronal cells were enriched in BPA-responsive genes, we obtained the list of RORA transcriptional targets from our previous study. Using chromatin immunoprecipitation (ChIP) followed by whole-genome promoter array (chip) analysis, we found that RORA1—a major isoform of RORA protein in the human brain—was recruited to as many as 2764 genomic locations corresponding to promoter regions of 2544 genes across the human genome [103]. The hypergeometric distribution analysis unveiled the strong associations between the BPA-responsive genes and the RORA1 transcription targets (Table S14), suggesting that RORA is involved in the effects of prenatal BPA exposure on gene expression in the prefrontal cortex. Notably, the BPA-responsive genes in male and female pups exhibited sex-specific associations with the targets of ASD-related transcription factors. This finding suggests that the BPA-mediated dysregulation of transcriptome profiles in the prefrontal cortex of male and female offspring may involve different sets of transcription factors.

### 2.6. Molecular Docking Analysis of BPA and ASD-Related Transcription Factors of Which the Targets Are Over-Represented among BPA-Responsive Genes

To predict whether BPA can directly interact with ASD-related transcription factors, we selected 10 transcription factors of which the targets are associated with BPA-responsive genes and manually curated them by the TRANSFAC Curated database. We performed molecular docking analysis with BPA molecules using Discovery Studio 2019 and AutoDock 4.2 software (Figure S2). The binding free energies of the complexes between BPA and each transcription factor were calculated. When available, the known ligands of each transcription factor were also used in molecular docking analysis for comparison. The molecular docking analysis revealed that BPA exhibited good binding affinity with AR, ESR1, RORA, SOX5, TCF4, and YY1 (Table 5). We also investigated the expression of genes encoding these transcription factor proteins (Figure S3A,B). The qRT-PCR analysis of Ar and Yy1 showed that the RNA levels of these transcription factors were not significantly changed in response to BPA, suggesting that BPA may interact with these transcription factor proteins and alter the activities of these transcription factors at the protein level without changing the RNA expression. 

**Figure 2:**
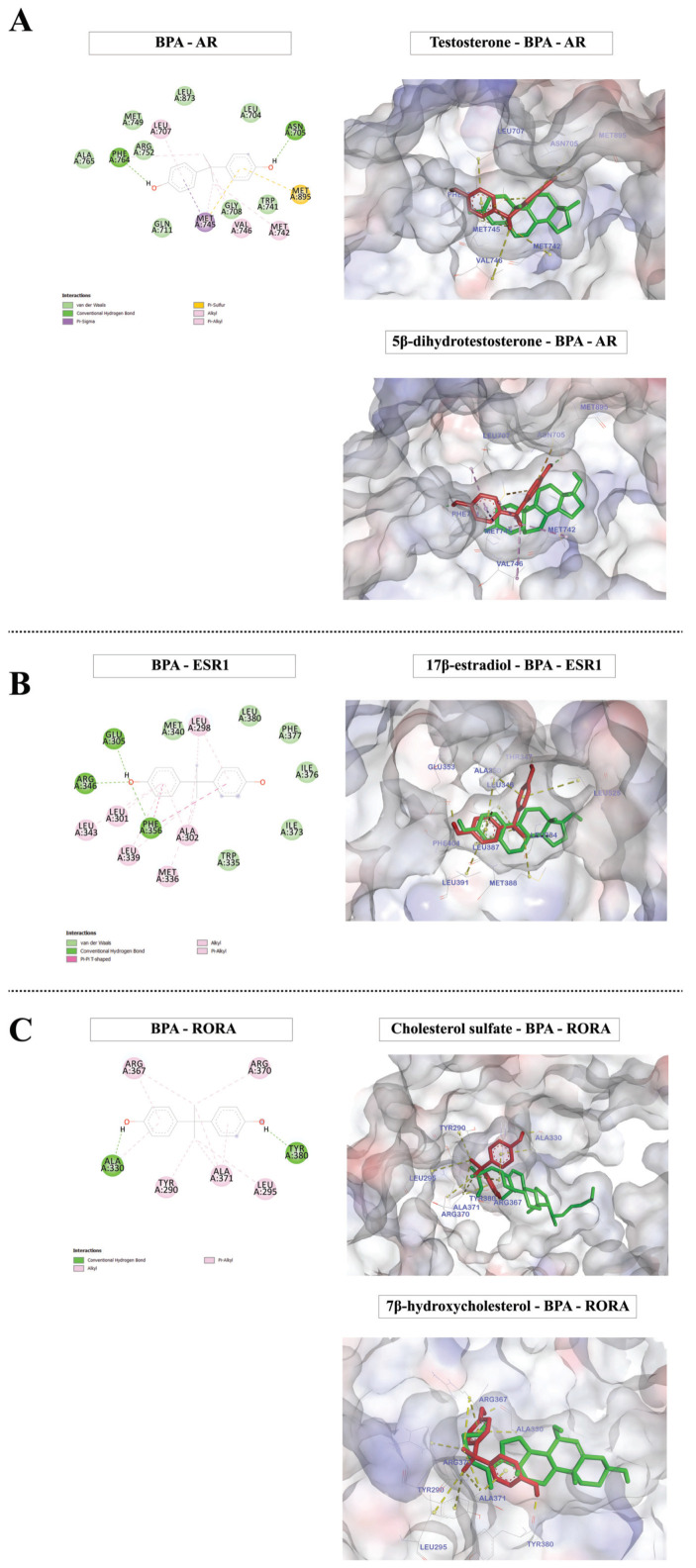
Molecular docking of BPA and AR/ESR1/RORA, of which the targets were over-represented among BPA-responsive genes in the offspring prefrontal cortex. The molecular docking between BPA (red) and androgen receptor (AR) (**A**), estrogen receptor alpha (ESR1) (**B**), and retinoic acid-related orphan receptor-alpha (RORA) (**C**) was performed using Discovery Studio 2019 and Autodock 4.2 software. The known ligands (green) of these transcription factors were also used for comparison.

Moreover, we found that BPA can bind to the ligand-binding domains of AR, ESR1, and RORA at sites close to the known ligands of these transcription factors (Figure 2). These results suggest that BPA may alter the transcription of their target genes in the prefrontal cortex of offspring by interacting with these transcription factors.

## 3. Discussion

**Figure 3:**
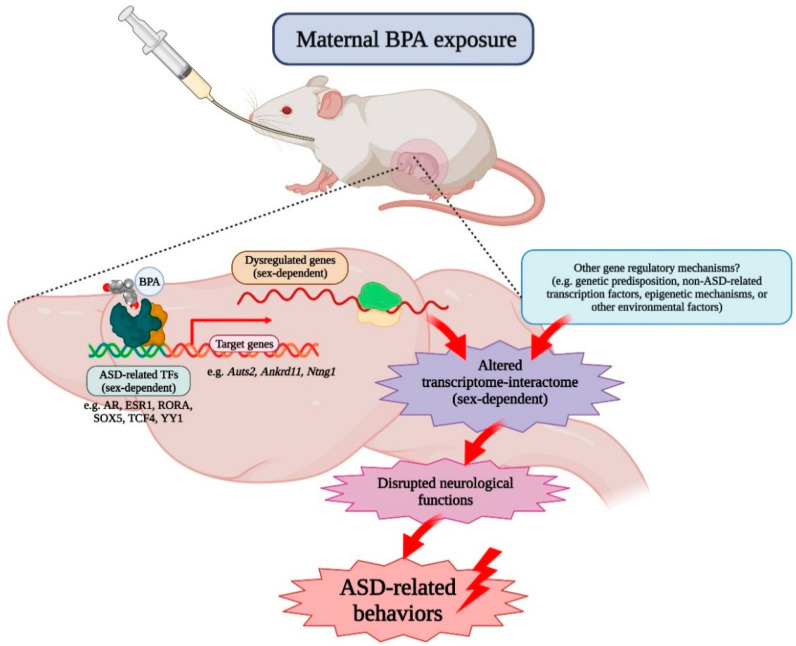
Schematic diagram illustrating a possible mechanism of prenatal BPA exposure on the offspring prefrontal cortex. We propose that maternal BPA exposure alters the transcriptome–interactome profiles in the prefrontal cortex of offspring in a sex-dependent manner through ASD-related transcription factors (e.g., AR, ESR1, RORA, SOX5, TCF4, and YY1), as well as other gene regulatory mechanisms (e.g., genetic predisposition, non-ASD-related transcription factors, epigenetic mechanisms, or other environmental factors). Disrupted transcriptome–interactome profiles lead to neuropathological conditions in the ASD brain and changes in behaviors that are controlled by the prefrontal cortex and known to be negatively impacted in ASD, such as social interaction and executive functions. (This figure was created with BioRender.com).

Although ASD has a high degree of heritability and genetic factors are known to play an important role in the etiology and susceptibility of the disorder [105,106,107], recent evidence has shown that up to 40–50% of variance in the genetic liability of ASD is determined by environmental factors [108,109,110,111,112]. Recent human studies have reported that in utero BPA exposure is associated with altered behaviors and neurological functions frequently found in ASD [113,114,115,116,117,118,119,120,121,122], prompting the theory that exposure to BPA may cause or increase the risk of the disorder. Several animal studies have investigated the effects of adult or prenatal BPA exposure on the brain and found that BPA dysregulates gene expression in multiple regions, including the prefrontal cortex [77], the hippocampus [15,78], the hypothalamus [78], the amygdala [80], and the cerebellum [79]. Although these brain regions are known to be impacted in people with ASD [123,124], it is still unclear how prenatal BPA exposure can cause pathological conditions in the brain and lead to behavioral impairments that are the hallmarks of ASD. Moreover, the effects of in utero BPA exposure on the transcriptome profiles of the offspring’s prefrontal cortex—a brain region responsible for executive functions and social behaviors known to be negatively impacted in ASD—have not been investigated. This is the first study to demonstrate that prenatal BPA exposure, even at the (NOAEL), alters the transcriptome–interactome profiles of genes in the offspring’s prefrontal cortex in a sex-dependent manner, possibly through ASD-related transcription factors (Figure 3). 

This finding is consistent with our previous studies, which observed the sex-specific effects of maternal BPA exposure on the transcriptome–interactome profiles of the offspring hippocampus [15,16,125]. Moreover, Thongkorn et al. found that the expression of several ASD candidate genes (e.g., *Mief2*, *Eif3h*, *Cux1*, and *Atp8a1*) in the hippocampus is dysregulated in response to prenatal BPA exposure and shows a sex-specific correlation with neuronal viability, neuritogenesis, and/or learning/memory [16]. The neuronal viability and neuronal density in the hippocampus and learning/memory are reduced only in the male offspring, while those in the females are not affected. In addition to the hippocampus, sex differences in the effects of prenatal BPA exposure on transcriptome profiles are also found in the hypothalamus and amygdala by other animal studies [78,80].

The gene ontology analysis by IPA software revealed that the BPA-responsive genes in the offspring’s prefrontal cortex were significantly associated with ASD and other disorders, including pervasive developmental disorder, schizophrenia spectrum disorder, movement disorders, and syndromic encephalopathy, all of which are known to be comorbid disorders of ASD [126,127,128,129]. A significant association of BPA-responsive genes and ASD was observed, both when male and female pups were combined into one group for each treatment and when each sex was analyzed separately. In addition to the results from the IPA analysis, the known ASD candidate genes available in the SFARI database were also over-represented in the lists of BPA-responsive genes. Moreover, DEGs in response to prenatal BPA exposure were also significantly associated with dysregulated genes in postmortem brain tissues from ASD cases. Strong associations were observed between BPA-responsive genes in the offspring’s prefrontal cortex and DEGs in the frontal and temporal cortex of ASD cases, while DEGs in the occipital lobe and cerebellum of ASD cases showed little or no association. The association of BPA-responsive genes and ASD was also found in the hippocampus of pups prenatally exposed to BPA [15]. Taken together, these findings suggest that prenatal BPA exposure may increase the risk of ASD by affecting multiple brain regions of the offspring simultaneously, but changes in the transcriptome profiles and their association with ASD may be specific to certain areas of the brain.

The biological function and canonical pathway analysis by IPA also showed that BPA-responsive genes were involved in neurological functions and pathways known to be disrupted in ASD, including axonal guidance signaling [130], PTEN signaling [131], synaptic long-term depression and potentiation [132,133], and Wnt/calcium signaling [134]. In addition, DEGs in the prefrontal cortex of pups prenatally exposed to BPA were also associated with “proliferation of neuronal cells”, “neuritogenesis”, and “neurotransmission”. Consistent with our findings, a recent study in mice reported that in utero BPA exposure suppresses the proliferation and differentiation of cortical neural progenitor cells during brain development. Moreover, synaptic formation and transmission in the cerebral cortex are also observed in mice prenatally exposed to BPA [135]. Interestingly, neural stem cells, neural progenitor cells, and neurons derived from ASD cases also exhibit abnormal proliferation, neurogenesis, and synaptogenesis [136,137]. Notably, BPA-responsive genes in the prefrontal cortex of male pups, but not female pups, are also significantly associated with androgen signaling and estrogen receptor signaling, both of which have been implicated in the male bias of ASD [7,138,139].

The interactome network analysis unveiled the interactions among DEGs in response to BPA. Interestingly, the *Lnpk*, *Kmt2a*, and *Neo1* genes are found to be the hub genes of DEGs in the prefrontal cortex of offspring. *Lnpk* or Lunapark, ER junction formation factor, is the gene responsible for shaping and stabilizing ER membrane proteins [140]. A study reported that three children who carried *Lnpk* gene mutations exhibited ASD-related neurodevelopmental defects, such as severe psychomotor delay, intellectual disability, hypotonia, and epilepsy [141]. *Kmt2a*, also known as the *Mll* gene, encodes a transcriptional coactivator that regulates the transcription of genes related to neurogenesis [142,143]. The loss of *Kmt2a* in forebrain neurons results in behavioral changes, such as increased anxiety, social behavior deficit, and impaired working memory, in an animal model [144,145]. In addition, de novo *Kmt2a* loss-of-function variants were found in people with neurodevelopmental disorders, including ASD [146]. *Neo1*, encoding neurogenin 1, is the hub gene of female DEGs. Neurogenin 1 is a receptor for Netrin-1, which is a protein involved in axon guidance signaling [147]. Moreover, *Neo1* is expressed near the cortical plate during embryogenesis and plays an essential role in controlling neuronal migration in the embryonic brain [147,148]. Moreover, *Neo1* controls NSC proliferation, neurogenesis, and synaptogenesis and is also involved in the expression of depressive-like behavior [149]. Genetic abnormalities in this gene, such as deletion, missense variants, and duplications, were observed in ASD cases [150]. The effects of prenatal BPA exposure on these genes in the offspring prefrontal cortex and their impacts on neurological functions and behaviors associated with ASD deserve further investigation.

To further investigate the effects of prenatal BPA exposure on genes associated with ASD in the prefrontal cortex, five ASD candidate genes (i.e., *Ntng1*, *Auts2*, *Ankrd11*, *Dock4,* and *Syne1*) from the SFARI database were selected for qRT-PCR analysis. When male and female pups were combined into one group for each treatment, the expression of *Ntng1* was significantly reduced, whereas *Auts2* was increased in the prefrontal cortex of pups prenatally exposed to BPA. The changes in the expression of *Ankrd11*, *Dock4*, and *Syne1* were not significant. When each sex of pups was analyzed separately, *Ntng1* was downregulated and *Ankrd11* was upregulated in female pups in response to prenatal BPA exposure. Although *Auts2* and *Ankrd11* expression tended to increase in the male BPA group, no significant change was observed. *Ntng1* or netrin G1 encodes a membrane protein that functions in axon and dendrite growth during brain development [151,152,153]. Netrin-G1 knockout mice show anxiety behaviors in the elevated plus-maze test and exhibited deficits in fear response behavior [154]. Missense mutations of *NTNG1* were found in children with ASD [155]. The disruption of the *NTNG1* gene from chromosome 1 abnormal rearrangement was detected in a female patient with Rett syndrome, a progressive neurodevelopmental disorder once characterized as a type of ASD [156]. *Auts2* (autism susceptibility candidate 2 or activator of transcription and developmental regulator AUTS2) has been implicated in neurodevelopment due to its abundant expression in the developing brain, including the frontal cortex and the hippocampus [157,158]. Both copy number variation (CNV) duplications and deletions of *AUTS2* were found in patients with developmental delay and ASD [159]. Several studies have also identified rare mutations in the *Auts2* gene with ASD susceptibility [160,161,162]. *Auts2* mutations cause abnormal cortical neuronal migration and neurite formation in the developing mouse brain [91]. The knockout or loss-of-function mutation of *Auts2* causes microcephaly [161], neuron reduction [163], increased excitatory synapses, and ASD-like behaviors [164]. *Ankrd11* is an ankyrin repeat domain-containing protein 11. *Ankrd11* is expressed in precursor cells and neurons of the developing cortex [93]. *Ankrd11* is associated with chromatin or transcription regulators that control the acetylation of histones and gene expression during the development of neurons. Several studies have shown associations of deletion or mutations of the *Ankrd11* gene with ASD [165,166]. Duplication involving *ANKRD11* was found in patients with KBG syndrome, which exhibits severe developmental delay and intellectual disability [167,168]. The downregulation of *Ankrd11* in developing murine or human cortical neural precursor cells causes decreased neural proliferation, reduced neurogenesis, and abnormal neuronal positioning [93]. Moreover, an animal model with downregulated *Ankrd11* in the brain displays abnormal locomotion activity, social interaction deficits, and repetitive behavior, all of which are ASD-related behaviors [93]. The role of these genes, specifically the downregulation of *Ntng1* and the upregulation of *Auts2* and *Ankrd11*, in the context of ASD and the underlying molecular mechanisms through which BPA regulates the expression of these genes should be further studied.

BPA can bind to several nuclear receptor proteins, including AR, ESR1, THRA, ERRG, and PPARG, which may affect downstream signaling and dysregulate cellular functions [96,97,98,169]. Several nuclear receptors and other types of transcription factors are associated with ASD [88,103,170,171,172]. In this study, we found that as many as 96 human transcription factors have been associated with ASD and listed in the SFARI database as ASD candidate genes. Among those, the targets of 34 transcription factors were available in the Harmonizome database. Interestingly, the targets of as many as 25 out of 34 transcription factors were significantly over-represented in the lists of BPA-responsive genes, suggesting that these transcription factors may be responsible for altered transcriptome profiles in the prefrontal cortex of pups prenatally exposed to BPA. AR, ESR1, and THRA, which are known to be BPA targets, and were also identified as significant transcription factors in this study. The association between BPA-responsive genes and the targets of RORA was further confirmed using the list of RORA1 targets in the human neuronal cell line SH-SY5Y identified by our previous study [103]. RORA1 targets were significantly enriched in the lists of BPA-responsive genes in the prefrontal cortex of male pups and female pups when compared to age/sex-matched control pups, suggesting that RORA1 is involved in the effects of BPA on dysregulation of genes in the offspring prefrontal cortex. ERRG and PPARG were not listed as ASD candidate genes in the SFARI database and thus were not used for hypergeometric analysis and molecular docking in this study. In addition to these transcription factors, we identified transcription factors, RORA, SOX5, TCF4, and YY1, as novel targets of BPA by molecular docking analysis. Although these transcription factors were obtained from human databases, all transcription factors are also conserved in rats according to the NCBI HomoloGene database (https://www.ncbi.nlm.nih.gov/homologene, accessed on 25 May 2020). It is noteworthy that *Ntng1* is known to be a transcriptional target of RORA [103], TCF4 [173], and YY1 [174]. *Auts2* is a transcriptional target of AR [175], SOX5 [101,102], TCF4 [173], and YY1 [174]. *Ankrd11* is a transcriptional target of TCF4 [173] and YY1 [174]. Both *Dock4* and *Syne1* are transcriptional targets of ESR1 [101,102] and YY1 [174]. Moreover, AR, ESR1, and RORA have been implicated in the sex bias of ASD [86,87,139]. Our previous study showed that male and female hormones differentially regulated the expression of RORA in the human neuronal cell line SH-SY5Y through AR and ESR1, respectively. Moreover, we found that RORA transcriptionally regulated aromatase and that the protein levels of RORA and aromatase were significantly reduced in the frontal cortex of people with ASD [86,176]. The direct interaction between BPA and these transcription factors, protein, and RNA expression of all ASD-related transcription factors, as well as its effects on the signaling mediated by these transcription factors, deserve further investigation.

## 4. Materials and Methods

### 4.1. Animal Husbandry and Treatment

Eight-week-old male and female Wistar Furth rats were obtained from the National Laboratory Animal Center (NLAC), Thailand. All animals were housed at the Chulalongkorn University Laboratory Animal Center (CULAC) under standard temperature (21 ± 1 °C) and humidity (30–70%) conditions in a 12-h light/dark cycle with food and RO-UV water available ad libitum. After mating, female rats (GD1; *n* = 6) were divided into two groups, i.e., the BPA treatment group and the control group. The weight of each rat was measured daily and used to calculate the amount of BPA or vehicle control needed to treat each rat. Animal treatment was performed as previously described [15]. For BPA treatment, BPA (Sigma-Aldrich, St. Louis, MO, USA) was dissolved in absolute ethanol (Merck Millipore, Burlington, MA, USA) to a final concentration of 250 mg/mL to make a stock BPA solution. Then, the stock solution was further diluted with corn oil to a final concentration of 5000 µg/kg·maternal BW of BPA to treat each rat. The vehicle control treatment was prepared by mixing absolute ethanol with corn oil in amounts equivalent to those used for preparing BPA. After mating, female rats were intragastrically administered by either BPA or vehicle control from GD1 until parturition. To prevent cross-contamination of the treatment conditions, the rats in the BPA and control groups were raised separately in individually ventilated cages in a biohazard containment housing system. Separate sets of stainless-steel needles and all consumable products were used for oral gavage. All reusable materials were cleaned with ethanol and rinsed with copious amounts of Milli-Q deionized water before use. All experimental procedures were approved by the Chulalongkorn University Animal Care and Use Committee (animal use protocols numbers: 1673007, 1773011, and 2073011), Chulalongkorn University.

### 4.2. Tissue Dissection

Male and female neonatal rat pups from independent litters (*n* = 3 pups/sex/treatment) were used for transcriptome profiling and qRT-PCR analysis. Prefrontal cortex tissues were identified and dissected under a Nikon SMZ18 Stereo Microscope (Nikon, Tokyo, Japan) as previously described with slight modifications [177]. According to the protocol by Guo et al. [178], rat pups (postnatal days (PNDs) 1–2) were deeply anesthetized by intraperitoneal injection of 100 mg/kg·BW sodium pentobarbital and euthanized by decapitation. The brain was quickly and carefully removed from the skull and placed in a prechilled cell culture dish containing ice-cold, freshly prepared 1× HBSS (Invitrogen, Waltham, MA, USA) containing 30 mM glucose (Sigma-Aldrich, Saint Louis, MO, USA), 2 mM HEPES (GE Healthcare Bio-Sciences, Piscataway, NJ, USA), and 26 mM NaHCO_3_ (Sigma-Aldrich, Saint Louis, MO, USA). The meninges were completely removed. The prefrontal cortex was dissected and immediately placed in a tube with RNA stabilization reagent (RNAlater) (Ambion, Austin, TX, USA) and stored at −80 °C according to the manufacturer’s protocol until use.

### 4.3. RNA Isolation

Total RNA was extracted and purified using the mirVana™ miRNA Isolation Kit (Thermo Fisher Scientific, Waltham, MA, USA) according to the manufacturer’s protocol. Briefly, prefrontal cortex tissues were lysed in a denaturing lysis buffer, which stabilized RNA and inactivated RNases. Prefrontal cortex tissue lysates were then subjected to acid-phenol:chloroform extraction to purify RNA and remove DNA. Ethanol was then added to the samples and passed through a filter cartridge containing a glass-fiber filter that immobilized the RNA. The filter was then washed three times, and finally, total RNA was eluted with a low ionic-strength solution. The purity of total RNA was assessed using a NanoDrop spectrophotometer (Thermo Fisher Scientific, Waltham, MA, USA) and quantified by using Invitrogen Qubit 2.0 Fluorometer (Thermo Fisher Scientific, Waltham, MA, USA). An Agilent 2100 BioAnalyzer (Agilent Technologies, Santa Clara, CA, USA) was used to determine RNA integrity. The results from the bioanalyzer were presented as the 28S:18S rRNA ratio and the RNA integrity number (RIN). The 28S:18S rRNA ratio was greater than 1.0, and the RIN was greater than 7.0 for all RNA samples.

### 4.4. Transcriptome Profiling Analysis

To identify DEGs in response to maternal BPA exposure, a transcriptome profiling analysis of total RNA isolated from the prefrontal cortex of neonatal rats from six independent litters prenatally exposed to BPA or vehicle control was performed by BGI Genomics Co., Ltd., China, using the Illumina HiSeq 4000 next-generation sequencing platform with 4 G reads (Illumina, San Diego, CA, USA) as previously described [15]. For RNA-seq analysis, RNA quality requirements were total RNA of ≥200 ng, RNA concentrations of ≥20 ng/µL, RINs of ≥7.0, and 28S/18S rRNA ratios of ≥1.0. Briefly, total RNA was treated with DNase I to remove DNA, and oligo(dT) treatment was used for mRNA isolation. Next, mRNA samples were fragmented by adding a fragmentation buffer, and reverse transcription was performed using mRNA fragments as templates. Short fragments were purified and resolved with an EB buffer for end repair and single nucleotide adenine addition. After that, the short fragments were connected with adapters, and suitable fragments were selected for PCR amplification. For quality control, an Agilent 2100 Bioanalyzer (Agilent Technologies, Santa Clara, CA, USA) and an ABI StepOnePlus Real-Time PCR System (Applied Biosystems, Waltham, MA, USA) were used in the quantification and qualification of the sample library. The library was sequenced using Illumina HiSeq 4000 (Illumina, San Diego, CA, USA). Subsequently, sequencing reads were filtered and subjected to quality control. Clean reads in a FASTQ file were mapped to the rat reference genome Rnor_6.0 (RefSeq ID: 1174938) using Bowtie 2 [15,179], and gene expression levels were then calculated using RSEM [180]. The transcriptome profiles between the BPA and the control groups were compared using a Poisson distribution. Comparisons were performed with all male and female pups with the same treatment condition combined into one group and separately for each sex. *p*-values were calculated using a Poisson distribution method. DEGs with a *p*-value of <0.05 and an FDR of <0.05 were considered statistically significant.

### 4.5. qRT-PCR Analysis

qRT-PCR analysis was performed to confirm the expression of five selected ASD candidate genes that were differentially expressed in response to prenatal BPA exposure. Reverse transcription was performed using a RevertAid First Strand cDNA Synthesis Kit (Thermo Scientific, Waltham, MA, USA) following the manufacturer’s protocol. Briefly, a total of 0.5 μg total RNA were mixed with 0.2 μg random hexamer primer and incubated at 65 °C for 5 min. After that, cDNA synthesis reagents consisting of 4 μL of a 5X Reaction Buffer, 1 μL of a RiboLock RNase Inhibitor (20 U/µL), 2 μL of 10 mM dNTP Mix, and 1 μL of RevertAid M-MuLV Reverse Transcriptase (200 U/µL) were added to the mixture and brought the total volume to 20 μL. Reverse transcription was performed by incubation at 25 °C for 5 min, followed by 42 °C for 60 min. The reaction was terminated by heating the solution to 70 °C for 5 min.

qPCR analysis was performed using AccuPower^®^ 2X GreenStar™ qPCR MasterMix (Bioneer, Daejeon, South Korea) according to the manufacturer’s instructions. Briefly, 1 μL of cDNA was mixed with 2X Greenstar Master Mix, a forward primer, a reverse primer, and nuclease-free water. Each sample was prepared in triplicate reactions. The reaction was then incubated in a Bio-Rad CFX Connect Real-Time PCR Detection System (Bio-Rad, Hercules, CA, USA). The PCR conditions were set as follows: an initial denaturing step at 95 °C for 15 min, 40 cycles of 95 °C for 10 s per cycle, and 30 s at 55 °C for annealing/extension. Melting curve analysis was set at 65 to 95 °C for product confirmation. The expression levels were calculated by the 2^−ΔΔCt^ method using the 18S ribosomal RNA (*Rn18s*) gene as an endogenous control. The primers used in this study were designed using the UCSC Genome Browser (https://genome.ucsc.edu/, accessed on 17 May 2019) [181], Ensembl (https://asia.ensembl.org/index.html, accessed on 17 May 2019) [182], and Primer3 software (http://bioinfo.ut.ee/primer3-0.4.0/, accessed on 17 May 2019) [183,184,185]. Forward and reverse primers were designed for rat *Auts2*, *Ankrd11*, *Ntng1*, *Dock4, Syne1*, and *Rn18s*. The sequences of the qPCR primers are shown in Table S15.

### 4.6. Prediction of Biological Functions, Disorders, Canonical Pathways, and Interactome Networks Associated with DEGs

Biological functions, disorders, canonical pathways, and interactome networks associated with DEGs were predicted using IPA (QIAGEN Inc., https://www.qiagenbioinformatics.com/products/ingenuity-pathway-analysis/, accessed on 25 May 2020) [89]. The list of DEGs overlapped with the list of genes experimentally validated to be associated with each function/disorder/canonical pathway in Ingenuity’s Knowledge Base database. Fisher’s exact test was then performed to calculate *p*-values, and a *p*-value of <0.05 was considered statistically significant.

### 4.7. Transcriptome Profiling Analysis of Postmortem Brain Tissues from ASD and Unaffected Individuals

To identify significantly DEGs in the brains of ASD cases, the transcriptome profiling data of postmortem brain tissues from ASD cases and typically developing people were obtained from the NCBI Gene Expression Omnibus (GEO) repository (http://www.ncbi.nlm.nih.gov/gds, accessed on 2 May 2020) [186] in a search performed on 2 May 2020, using “postmortem brain and autism” as a keyword. The details of the transcriptome profiling datasets used in this study are shown in Table S9. Transcriptome profiling data from each study were then analyzed separately according to the brain region by using Multiple Experiment Viewer (MeV) software (http://mev.tm4.org/86, accessed on 9 May 2020) [187]. To identify DEGs in ASD brain tissues, two-tailed t-tests with adjusted Bonferroni correction were performed. Then, the lists of DEGs from postmortem brain tissues overlapped with the list of BPA-responsive DEGs in our study. The significant associations between DEGs in the brains of ASD cases and BPA-responsive genes were determined by using the hypergeometric distribution calculator program in the Keisan Online Calculator package (http://keisan.casio.com/exec/system/1180573201, accessed on 18 May 2020). Four parameters in the hypergeometric distribution calculator were the number of overlapping genes, the number of BPA-responsive genes, the number of DEGs from the postmortem brain tissues that were detected in the rat prefrontal cortex, and the number of genes detectable in the prefrontal cortex by RNA-seq analysis. A hypergeometric *p*-value of <0.05 was considered statistically significant.

### 4.8. Molecular Docking

To determine whether BPA can directly interact with transcription factors of which the target genes are associated with BPA-responsive genes. BPA structure was obtained from PubChem Open Chemistry Database (NIH, USA, https://pubchem.ncbi.nlm.nih.gov, accessed on 25 May 2020), and protein structures were obtained from RCSB Protein Data Bank (PDB) (https://www.rcsb.org/, accessed on 25 May 2020). The criteria for choosing transcription factor structures from the PDB database for docking were as follows: (i) the resolution was less than 3 Angstrom; and (ii) the structure was discovered by X-ray diffraction. For transcription factors of which the structures are not available in the PDB database, the structures were obtained from the Alphafold Protein Structure Database (https://alphafold.ebi.ac.uk/, accessed on 25 May 2020) [188]. The Discovery Studio Visualizer program (BIOVIA, San Diego, CA, USA, http://www.3dsbiovia.com/products/collaborative-science/biovia-discovery-studio/visualization-download.php, accessed on 30 May 2020) was used to remove crystal water molecules and to add partial charges to each atom. Then, the molecular docking between BPA, known ligands, and transcription factors was performed using Autodock 4 and AutodockTools 4 [189]. The binding abilities between BPA or known ligands and each transcription factor were evaluated and were shown as Gibbs free energy (ΔG). For each pair of BPA/ligands and a transcription factor, the binding free energy was averaged from three independent runs.

## 5. Conclusions

Our transcriptomic profiling analysis revealed that in utero BPA exposure caused the sex-dependent dysregulation of transcriptome-interactome profiles in the prefrontal cortex of neonatal rat pups. BPA-responsive genes were associated with ASD and related neurological functions and pathways. Moreover, known ASD candidate genes and genes disrupted in the brains of ASD cases were significantly enriched in the lists of BPA-responsive genes. In addition, BPA-responsive genes are known targets of several transcription factors, including AR, ESR1, and RORA, which have been linked to the pathobiology or sex bias of ASD. Molecular docking predicted that BPA may directly interact with these transcription factors, many of which are novel targets for BPA, including RORA, SOX5, TCF4, and YY1. Taken together, these findings suggest that in utero BPA exposure may increase the risk of ASD by impacting ASD-related genes in the offspring’s prefrontal cortex, possibly through sex-specific transcription factors associated with the disorder. A better understanding of prenatal BPA exposure effects and the underlying molecular mechanisms may lead to increased awareness and development of molecular targets for treatment in the future.

## Figures and Tables

**Table 1:** Neurological disorders associated with differentially expressed genes (DEGs) in the offspring’s prefrontal cortex in response to prenatal bisphenol A (BPA) exposure. The lists show the DEGs in the prefrontal cortex of rat pups prenatally exposed to BPA when both male and female pups were combined into one group for each treatment and each sex of pups was analyzed. *p*-value < 0.05 was considered as significant. NS, not significant. NA, not available.

Diseases or Disorders	*p*-Value (Number of Genes)		
	Both Sexes	Male	Female
Autism or intellectual disability	4.11 × 10^−11^ (233)	1.89 × 10^−13^ (128)	2.31 × 10^−5^ (105)
Schizophrenia spectrum disorder	NS	3.64 × 10^−8^ (128)	1.75 × 10^−9^ (139)
Mood disorders	4.82 × 10^−10^ (227)	3.40 × 10^−8^ (111)	1.27 × 10^−6^ (110)
Pervasive developmental disorder	2.05 × 10^−7^ (107)	4.92 × 10^−5^ (50)	NA
Disorder of basal ganglia	1.15 × 10^−23^ (516)	1.60 × 10^−12^ (186)	1.42 × 10^−11^ (191)
Movement disorders	4.36 × 10^−37^ (760)	5.32 × 10^−16^ (234)	1.96 × 10^−14^ (239)
Amyotrophic lateral sclerosis	NA	1.84 × 10^−6^ (59)	3.57 × 10^−9^ (69)
Alzheimer disease	6.74 × 10^−19^ (347)	3.35 × 10^−11^ (163)	1.28 × 10^−11^ (172)
Huntington’s disease	6.54 × 10^−19^ (388)	6.67 × 10^−9^ (126)	2.30 × 10^−10^ (137)
Syndromic encephalopathy	3.77 × 10^−21^ (358)	1.49 × 10^−7^ (75)	5.82 × 10^−6^ (73)

**Table 2:** Associations between the BPA-responsive genes and the known ASD candidate genes from the SFARI database. Hypergeometric distribution analysis was performed to determine the associations between the lists of BPA-responsive genes in the offspring’s prefrontal cortex and the known ASD candidate genes from the SFARI database. The scores determined by the SFARI database indicate confidence levels for each group of ASD candidate genes. Statistically significant associations were determined by hypergeometric distribution analysis (*p*-value < 0.05).

Gene List Category (No. of Genes)	Both Sexes		Male		Female	
	No. of Target Genes Detected in the Rat Frontal Cortex	No. of Overlapping Genes (*p*-Value)	No. of Target Genes Detected in the Rat Frontal Cortex	No. of Overlapping Genes (*p*-Value)	No. of Target Genes Detected in the Rat Frontal Cortex	No. of Overlapping Genes (*p*-Value)
All genes (986)	835	**408 (4.44 × 10^−3^)**	847	**243 (6.64 × 10^−17^)**	843	**228 (1.70 × 10^−10^)**
Syndromic (143)	130	66 (0.085)	133	**49 (7.32 × 10^−8^)**	129	**38 (1.54 × 10^−3^)**
Score 1 High confidence (25)	23	10 (0.615)	23	**10 (3.27 × 10^−3^)**	23	**11 (1.25 × 10^−3^)**
Score 2 Strong candidate (59)	54	24 (0.552)	55	**18 (4.45 × 10^−3^)**	55	**17 (1.77 × 10^−2^)**
Score 3 Suggestive evidence (176)	159	70 (0.572)	163	**53 (2.11 × 10^−6^)**	159	**52 (1.10 × 10^−5^)**
Score 4 Minimal evidence (406)	321	**162 (1.62 × 10^−2^)**	322	**90 (1.70 × 10^−6^)**	324	**84 (4.84 × 10^−4^)**
Score 5 Hypothesized (157)	133	68 (0.071)	135	28 (0.186)	136	30 (0.164)

**Table 3:** Associations between BPA-responsive genes and DEGs from postmortem brain tissues of people with ASD. Hypergeometric distribution analysis was performed to determine the associations between the lists of BPA-responsive genes in the offspring’s prefrontal cortex and genes differentially expressed in the brain of ASD cases. Statistically significant associations were determined by hypergeometric distribution analysis (*p*-value < 0.05).

Studies (Year)	Brain Region	Both Sexes (6284 Genes)			Male (2565 Genes)			Female (2706 Genes)		
		No. of DEGs Detected in the Rat Frontal Cortex	No. of Overlapping Genes	*p*-Value	No. of DEGs Detected in the Rat Frontal Cortex	No. of Overlapping Genes	*p*-Value	No. of DEGs Detected in the Rat Frontal Cortex	No. of Overlapping Genes	*p*-Value
Parikshak et al. (2015)	Frontal and temporal cortex	954	462	**5.61 × 10^−3^**	956	221	**2.71 × 10^−6^**	958	219	**2.28 × 10^−4^**
Voineagu et al. (2011)	Frontal cortex (BA9, BA44/45)	384	177	0.269	383	89	**2.22 × 10^−3^**	385	86	**0.029**
Chow et al. (2012)	Prefrontal cortex	71	36	0.172	76	24	**1.94 × 10^−3^**	73	16	0.264
Garbett et. al. (2008)	Temporal cortex	101	58	**5.72 × 10^−3^**	102	21	0.238	101	29	**7.72 × 10^−3^**
Voineagu et al. (2011)	Temporal cortex (BA41/42, 22)	545	248	0.318	546	117	**8.98 × 10^−3^**	539	120	**0.013**
Ginsberg et al. (2012)	Occipital lobe (BA19)	269	116	0.690	270	60	**0.026**	277	56	0.245
Ginsberg et al. (2012)	Cerebellum	749	307	0.977	744	150	**0.029**	746	151	0.108
Voineagu et al. (2011)	Cerebellum; vermis	57	24	0.685	58	12	0.309	58	11	0.514

**Table 4:** ASD-related transcription factors of which the target genes are over-represented among BPA-responsive genes in the offspring prefrontal cortex. Hypergeometric distribution analyses were performed to determine the associations between the BPA-responsive genes in the offspring prefrontal cortex and the lists of target genes of each autism-related transcription factor that are manually curated and available in the TRANSFAC Curated database. A *p*-value of <0.05 is considered as significant.

Transcription Factors	Both Sexes (6284 Genes)		Male (2565 Genes)		Female (2706 Genes)	
	No. of Target Genes Detected in the Rat Frontal Cortex	No. of Overlapping Genes (*p*-Value)	No. of Target Genes Detected in the Rat Frontal Cortex	No. of Overlapping Genes (*p*-Value)	No. of Target Genes Detected in the Rat Frontal Cortex	No. of Overlapping Genes (*p*-Value)
AR	609	274 (0.403)	614	**140 (3.56 × 10^−4^)**	618	**145 (8.48 × 10^−4^)**
CUX1	360	161 (0.475)	371	77 (0.056)	366	**82 (3.07 × 10^−2^)**
EGR2	173	80 (0.342)	170	37 (0.087)	171	**42 (2.74 × 10^−2^)**
ESR1	372	178 (0.098)	380	**81 (2.93 × 10^−2^)**	378	82 (0.060)
MTF1	211	103 (0.111)	220	**54 (4.78 × 10^−3^)**	213	**50 (3.79 × 10^−2^)**
PAX5	137	67 (0.165)	137	26 (0.355)	138	32 (0.094)
PAX6	85	42 (0.206)	85	15 (0.529)	84	16 (0.489)
POU3F2	435	189 (0.679)	437	**96 (8.54 × 10^−3^)**	441	90 (0.156)
RORA	264	109 (0.864)	266	**63 (5.84 × 10^−3^)**	266	**69 (1.45 × 10^−3^)**
SMAD4	196	86 (0.589)	195	39 (0.200)	194	31 (0.839)
SOX5	219	104 (0.198)	214	**56 (8.80 × 10^−4^)**	221	**55 (1.01 × 10^−2^)**
STAT1	304	130 (0.741)	305	64 (0.063)	306	64 (0.148)
TCF4	375	**187 (1.83 × 10^−2^)**	379	**96 (6.38 × 10^−5^)**	377	**94 (9.40 × 10^−4^)**
YY1	633	259 (0.969)	645	**145 (5.38 × 10^−4^)**	634	114 (0.641)

**Table 5:** Molecular docking of ASD-related transcription factors of which the targets were over-represented in BPA-responsive genes and BPA. The molecular docking between ASD-related transcription factors of which the targets were enriched among BPA-responsive genes in the offspring frontal cortex was performed using Discovery Studio 2019 and Autodock 4.2 software. The mean value and standard deviation of binding free energy for each pair of transcription factor and BPA or known ligand were calculated from triplicates. NA, not available.

Protein ID	TFs	Name	Known Ligand	Mean Binding Free Energy ± SD (kcal/mol)	
				Known Ligand	BPA Ligand
PDB:2AM9	AR	Androgen receptor	Testosterone	−11.17 ± 0.00	−8.98 ± 0.01
			5β-dihydrotestosterone	−11.07 ± 0.00	
PDB: 1A52	ESR1	Estrogen receptor alpha	17β-estradiol	−9.96 ± 0.00	−7.49 ± 0.00
PDB:1S0X	RORA	Retinoic acid-related orphan receptor-alpha	Cholesterol sulfate [104]	−12.64 ± 0.18	−7.47 ± 0.04
			7β-hydroxycholesterol [104]	−11.05 ± 0.16	
PDB:1I11	SOX5	SRY-box 5 (DNA-binding domain)	NA	NA	−5.85 ± 0.01
PDB:2KWF	TCF4	Transcription factor 4	NA	NA	−5.75 ± 0.04
PDB:1UBD	YY1	Yin Yang 1 transcriptional repressor protein (Zinc-finger domain)	NA	NA	−5.68 ± 0.02
Alphafold ID: Q14872	MTF1	Metal regulatory transcription factor 1	NA	NA	−4.33 ± 0.05
Alphafold ID: P20265	POU3F2	POU domain, class 3, and transcription factor 2	NA	NA	−4.33 ± 0.38
Alphafold ID: P39880	CUX1	Homeobox protein cut-like 1	NA	NA	−4.15 ± 0.37
Alphafold ID: P11161	EGR2	E3 SUMO-protein ligase EGR2	NA	NA	−3.66 ± 0.14

## Data Availability

The transcriptome profiling data used in this study have been published in the NCBI GEO DataSet database (GSE28475, GSE28521, and GSE38322). The RNA-seq data will be made publicly available in the GEO upon the acceptance of this manuscript for publication.

## Supplementary Materials

The following are available online at https://www.mdpi.com/article/10.3390/ijms222413201/s1.
