# Supplementary material for: Autism-Related Transcription Factors Underlying the Sex-Specific Effects of Prenatal Bisphenol A Exposure on Transcriptome-Interactome Profiles in the Offspring Prefrontal Cortex
Source: Int J Mol Sci. 2021 Dec 8;22(24):13201. doi: 10.3390/ijms222413201 (PMC8708761; doi:10.3390/ijms222413201)
Supplement: Supplementary file 1 [file ijms-22-13201-s001.zip › Table S13.pdf]

Table S13: Hypergeometric distribution analysis of 34 ASD-related transcription factors and BPA-responsive genes in the offspring frontal cortex. *P*-value < 0.05 is considered as significant.

NA = no data available.

TFs	Databases	Both sexes (6,284 genes)		Male (2,565 genes)		Female (2,706 genes)	
		# Target genes detected in rat frontal cortex	# Overlapping genes (*p* value)	# Target genes detected in rat frontal cortex	# Overlapping genes (*p* value)	# Target genes detected in rat frontal cortex	# Overlapping genes (*p* value)
AR	CHEA	4,565	2,169 (1.94E-07)	4,602	998 (2.55E-19)	4,628	1,053 (1.67E-19)
AR	ENCODE	NA	NA	NA	NA	NA	NA
AR	JASPAR Predicted	NA	NA	NA	NA	NA	NA
AR	MotifMap Predicted	22	6 (0.970)	22	6 (0.173)	24	6 (0.274)
AR	TRANSFAC Curated	609	274 (0.403)	614	140 (3.56E-04)	618	145 (8.48E-04)
AR	TRANSFAC Predicted	NA	NA	NA	NA	NA	NA
BCL11A	CHEA	NA	NA	NA	NA	NA	NA
BCL11A	ENCODE	1,606	734 (0.143)	1,606	302 (0.075)	1,606	298 (0.469)
BCL11A	JASPAR Predicted	NA	NA	NA	NA	NA	NA
BCL11A	MotifMap Predicted	NA	NA	NA	NA	NA	NA
BCL11A	TRANSFAC Curated	NA	NA	NA	NA	NA	NA
BCL11A	TRANSFAC Predicted	NA	NA	NA	NA	NA	NA
CTCF	CHEA	1,243	524 (0.957)	1,249	221 (0.431)	1,247	221 (0.769)
CTCF	ENCODE	12,785	5,831 (1.29E-18)	12,888	2,442 (1.96E-43)	12,955	2,547 (9.62E-29)
CTCF	JASPAR Predicted	15	7 (0.530)	17	3 (0.592)	15	3 (0.542)
CTCF	MotifMap Predicted	760	362 (3.70E-02)	764	152 (4.13E-02)	760	154 (0.103)
CTCF	TRANSFAC Curated	NA	NA	NA	NA	NA	NA
CTCF	TRANSFAC Predicted	NA	NA	NA	NA	NA	NA
CUX1	CHEA	1,922	855 (0.488)	1,935	366 (4.11E-02)	1,940	370 (0.236)
CUX1	ENCODE	7,490	3,275 (0.964)	7,459	1,373 (1.44E-03)	7,483	1,392 (0.331)
CUX1	JASPAR Predicted	NA	NA	NA	NA	NA	NA
CUX1	MotifMap Predicted	NA	NA	NA	NA	NA	NA
CUX1	TRANSFAC Curated	360	161 (0.475)	371	77 (0.056)	366	82 (3.07E-02)
CUX1	TRANSFAC Predicted	NA	NA	NA	NA	NA	NA

TFs	Databases	Both sexes (6,284 genes)		Male (2,565 genes)		Female (2,706 genes)	
		# Target genes detected in rat frontal cortex	# Overlapping genes (*p* value)	# Target genes detected in rat frontal cortex	# Overlapping genes (*p* value)	# Target genes detected in rat frontal cortex	# Overlapping genes (*p* value)
EGR2	CHEA	NA	NA	NA	NA	NA	NA
EGR2	ENCODE	NA	NA	NA	NA	NA	NA
EGR2	JASPAR Predicted	NA	NA	NA	NA	NA	NA
EGR2	MotifMap Predicted	NA	NA	NA	NA	NA	NA
EGR2	TRANSFAC Curated	173	80 (0.342)	170	37 (0.087)	171	42 (2.74E-02)
EGR2	TRANSFAC Predicted	NA	NA	NA	NA	NA	NA
ERG	CHEA	2,363	1,041 (0.664)	2,347	438 (0.054)	2,380	430 (0.713)
ERG	ENCODE	NA	NA	NA	NA	NA	NA
ERG	JASPAR Predicted	NA	NA	NA	NA	NA	NA
ERG	MotifMap Predicted	NA	NA	NA	NA	NA	NA
ERG	TRANSFAC Curated	NA	NA	NA	NA	NA	NA
ERG	TRANSFAC Predicted	NA	NA	NA	NA	NA	NA
ESR1	CHEA	1,842	927 (2.90E-08)	1,855	424 (1.50E-10)	1,855	474 (2.74E-16)
ESR1	ENCODE	1,196	555 (0.080)	1,209	227 (0.116)	1,208	253 (1.19E-02)
ESR1	JASPAR Predicted	1,084	478 (0.603)	1,090	185 (0.690)	1,102	200 (0.621)
ESR1	MotifMap Predicted	18	11 (0.118)	19	4 (0.431)	18	6 (0.098)
ESR1	TRANSFAC Curated	372	178 (0.098)	380	81 (2.93E-02)	378	82 (0.060)
ESR1	TRANSFAC Predicted	NA	NA	NA	NA	NA	NA
ESR2	CHEA	348	169 (0.065)	345	70 (0.095)	346	80 (1.60E-02)
ESR2	ENCODE	NA	NA	NA	NA	NA	NA
ESR2	JASPAR Predicted	47	20 (0.656)	49	11 (0.227)	48	11 (0.263)
ESR2	MotifMap Predicted	30	17 (0.122)	30	8 (0.140)	30	9 (0.087)
ESR2	TRANSFAC Curated	NA	NA	NA	NA	NA	NA
ESR2	TRANSFAC Predicted	46	20 (0.608)	44	13 (3.39E-02)	44	14 (2.32E-02)
ESRRB	CHEA	1,169	531 (0.247)	1,171	245 (8.75E-04)	1,170	250 (4.71E-03)
ESRRB	ENCODE	NA	NA	NA	NA	NA	NA
ESRRB	JASPAR Predicted	46	18 (0.808)	46	5 (0.923)	46	8 (0.634)

TFs	Databases	Both sexes (6,284 genes)		Male (2,565 genes)		Female (2,706 genes)	
		# Target genes detected in rat frontal cortex	# Overlapping genes (*p* value)	# Target genes detected in rat frontal cortex	# Overlapping genes (*p* value)	# Target genes detected in rat frontal cortex	# Overlapping genes (*p* value)
ESRRB	MotifMap Predicted	268	118 (0.576)	266	52 (0.206)	265	62 (2.46E-02)
ESRRB	TRANSFAC Curated	NA	NA	NA	NA	NA	NA
ESRRB	TRANSFAC Predicted	NA	NA	NA	NA	NA	NA
FOXP1	CHEA	2,961	1,301 (0.734)	2,964	556 (2.22E-02)	2,981	569 (0.167)
FOXP1	ENCODE	NA	NA	NA	NA	NA	NA
FOXP1	JASPAR Predicted	NA	NA	NA	NA	NA	NA
FOXP1	MotifMap Predicted	NA	NA	NA	NA	NA	NA
FOXP1	TRANSFAC Curated	NA	NA	NA	NA	NA	NA
FOXP1	TRANSFAC Predicted	NA	NA	NA	NA	NA	NA
FOXP2	CHEA	1,417	653 (0.098)	1,421	288 (2.34E-03)	1,436	297 (1.29E-02)
FOXP2	ENCODE	8,491	3,772 (0.513)	8,451	1,662 (1.73E-16)	8,467	1,689 (2.70E-08)
FOXP2	JASPAR Predicted	NA	NA	NA	NA	NA	NA
FOXP2	MotifMap Predicted	NA	NA	NA	NA	NA	NA
FOXP2	TRANSFAC Curated	NA	NA	NA	NA	NA	NA
FOXP2	TRANSFAC Predicted	NA	NA	NA	NA	NA	NA
GTF2I	CHEA	NA	NA	NA	NA	NA	NA
GTF2I	ENCODE	NA	NA	NA	NA	NA	NA
GTF2I	JASPAR Predicted	NA	NA	NA	NA	NA	NA
GTF2I	MotifMap Predicted	NA	NA	NA	NA	NA	NA
GTF2I	TRANSFAC Curated	NA	NA	NA	NA	NA	NA
GTF2I	TRANSFAC Predicted	163	77 (0.258)	169	31 (0.415)	168	31 (0.532)
HOXA1	CHEA	NA	NA	NA	NA	NA	NA
HOXA1	ENCODE	NA	NA	NA	NA	NA	NA
HOXA1	JASPAR Predicted	NA	NA	NA	NA	NA	NA
HOXA1	MotifMap Predicted	3	1 (0.828)	3	1 (0.438)	3	1 (0.458)
HOXA1	TRANSFAC Curated	NA	NA	NA	NA	NA	NA
HOXA1	TRANSFAC Predicted	NA	NA	NA	NA	NA	NA

TFs	Databases	Both sexes (6,284 genes)		Male (2,565 genes)		Female (2,706 genes)	
		# Target genes detected in rat frontal cortex	# Overlapping genes (*p* value)	# Target genes detected in rat frontal cortex	# Overlapping genes (*p* value)	# Target genes detected in rat frontal cortex	# Overlapping genes (*p* value)
KDM5B	CHEA	2,999	1,313 (0.795)	3,013	650 (4.74E-11)	3,009	651 (3.91E-07)
KDM5B	ENCODE	9,923	4,419 (0.358)	9,925	1,905 (6.42E-16)	9,968	1,948 (3.66E-07)
KDM5B	JASPAR Predicted	NA	NA	NA	NA	NA	NA
KDM5B	MotifMap Predicted	NA	NA	NA	NA	NA	NA
KDM5B	TRANSFAC Curated	NA	NA	NA	NA	NA	NA
KDM5B	TRANSFAC Predicted	NA	NA	NA	NA	NA	NA
MBD4	CHEA	NA	NA	NA	NA	NA	NA
MBD4	ENCODE	4,816	2,083 (0.979)	4,796	849 (0.320)	4,792	831 (0.993)
MBD4	JASPAR Predicted	NA	NA	NA	NA	NA	NA
MBD4	MotifMap Predicted	NA	NA	NA	NA	NA	NA
MBD4	TRANSFAC Curated	NA	NA	NA	NA	NA	NA
MBD4	TRANSFAC Predicted	NA	NA	NA	NA	NA	NA
MEF2C	CHEA	NA	NA	NA	NA	NA	NA
MEF2C	ENCODE	690	313 (0.320)	688	125 (0.329)	687	122 (0.701)
MEF2C	JASPAR Predicted	NA	NA	NA	NA	NA	NA
MEF2C	MotifMap Predicted	NA	NA	NA	NA	NA	NA
MEF2C	TRANSFAC Curated	NA	NA	NA	NA	NA	NA
MEF2C	TRANSFAC Predicted	NA	NA	NA	NA	NA	NA
MTF1	CHEA	NA	NA	NA	NA	NA	NA
MTF1	ENCODE	NA	NA	NA	NA	NA	NA
MTF1	JASPAR Predicted	NA	NA	NA	NA	NA	NA
MTF1	MotifMap Predicted	NA	NA	NA	NA	NA	NA
MTF1	TRANSFAC Curated	211	103 (0.111)	220	54 (4.78E-03)	213	50 (3.79E-02)
MTF1	TRANSFAC Predicted	1,093	495 (0.286)	1,089	228 (1.27E-03)	1,104	225 (4.85E-02)
NFIA	CHEA	NA	NA	NA	NA	NA	NA
NFIA	ENCODE	NA	NA	NA	NA	NA	NA
NFIA	JASPAR Predicted	NA	NA	NA	NA	NA	NA

TFs	Databases	Both sexes (6,284 genes)		Male (2,565 genes)		Female (2,706 genes)	
		# Target genes detected in rat frontal cortex	# Overlapping genes (*p* value)	# Target genes detected in rat frontal cortex	# Overlapping genes (*p* value)	# Target genes detected in rat frontal cortex	# Overlapping genes (*p* value)
NFIA	MotifMap Predicted	NA	NA	NA	NA	NA	NA
NFIA	TRANSFAC Curated	NA	NA	NA	NA	NA	NA
NFIA	TRANSFAC Predicted	954	450 (4.19E-02)	969	159 (0.830)	971	177 (0.590)
NR2F1	CHEA	NA	NA	NA	NA	NA	NA
NR2F1	ENCODE	NA	NA	NA	NA	NA	NA
NR2F1	JASPAR Predicted	714	311 (0.698)	719	147 (1.95E-02)	728	153 (3.94E-02)
NR2F1	MotifMap Predicted	27	8 (0.962)	26	5 (0.486)	27	6 (0.380)
NR2F1	TRANSFAC Curated	NA	NA	NA	NA	NA	NA
NR2F1	TRANSFAC Predicted	594	268 (0.380)	599	115 (0.142)	609	118 (0.291)
OTX1	CHEA	NA	NA	NA	NA	NA	NA
OTX1	ENCODE	NA	NA	NA	NA	NA	NA
OTX1	JASPAR Predicted	NA	NA	NA	NA	NA	NA
OTX1	MotifMap Predicted	1	1 (0.444)	1	1 (0.175)	1	1 (0.185)
OTX1	TRANSFAC Curated	NA	NA	NA	NA	NA	NA
OTX1	TRANSFAC Predicted	NA	NA	NA	NA	NA	NA
PAX5	CHEA	NA	NA	NA	NA	NA	NA
PAX5	ENCODE	9,026	3,961 (0.960)	8,969	1,715 (2.26E-11)	9,014	1,693 (0.105)
PAX5	JASPAR Predicted	4	3 (0.234)	5	1 (0.617)	4	1 (0.558)
PAX5	MotifMap Predicted	NA	NA	NA	NA	NA	NA
PAX5	TRANSFAC Curated	137	67 (0.165)	137	26 (0.355)	138	32 (0.094)
PAX5	TRANSFAC Predicted	NA	NA	NA	NA	NA	NA
PAX6	CHEA	661	304 (0.215)	651	131 (4.08E-02)	662	139 (4.89E-02)
PAX6	ENCODE	NA	NA	NA	NA	NA	NA
PAX6	JASPAR Predicted	15	7 (0.530)	17	3 (0.592)	15	3 (0.542)
PAX6	MotifMap Predicted	NA	NA	NA	NA	NA	NA
PAX6	TRANSFAC Curated	85	42 (0.206)	85	15 (0.529)	84	16 (0.489)
PAX6	TRANSFAC Predicted	97	49 (0.134)	98	16 (0.660)	98	22 (0.185)

TFs	Databases	Both sexes (6,284 genes)		Male (2,565 genes)		Female (2,706 genes)	
		# Target genes detected in rat frontal cortex	# Overlapping genes (*p* value)	# Target genes detected in rat frontal cortex	# Overlapping genes (*p* value)	# Target genes detected in rat frontal cortex	# Overlapping genes (*p* value)
PITX1	CHEA	NA	NA	NA	NA	NA	NA
PITX1	ENCODE	NA	NA	NA	NA	NA	NA
PITX1	JASPAR Predicted	NA	NA	NA	NA	NA	NA
PITX1	MotifMap Predicted	1	0 (1.000)	1	0 (1.000)	1	0 (1.000)
PITX1	TRANSFAC Curated	NA	NA	NA	NA	NA	NA
PITX1	TRANSFAC Predicted	1,053	490 (0.081)	1,059	170 (0.906)	1,064	187 (0.791)
POU3F2	CHEA	1,097	519 (2.46E-02)	1,115	249 (9.68E-06)	1,121	246 (1.20E-03)
POU3F2	ENCODE	NA	NA	NA	NA	NA	NA
POU3F2	JASPAR Predicted	NA	NA	NA	NA	NA	NA
POU3F2	MotifMap Predicted	3	1 (0.828)	3	0 (1.000)	3	0 (1.000)
POU3F2	TRANSFAC Curated	435	189 (0.679)	437	96 (8.54E-03)	441	90 (0.156)
POU3F2	TRANSFAC Predicted	NA	NA	NA	NA	NA	NA
RORA	CHEA	NA	NA	NA	NA	NA	NA
RORA	ENCODE	NA	NA	NA	NA	NA	NA
RORA	JASPAR Predicted	NA	NA	NA	NA	NA	NA
RORA	MotifMap Predicted	2	1 (0.691)	2	1 (0.319)	2	0 (1.000)
RORA	TRANSFAC Curated	264	109 (0.864)	266	63 (5.84E-03)	266	69 (1.45E-03)
RORA	TRANSFAC Predicted	NA	NA	NA	NA	NA	NA
SETDB1	CHEA	2,899	1,330 (4.10E-02)	2,916	609 (6.20E-08)	2,946	649 (2.20E-08)
SETDB1	ENCODE	6,702	2,921 (0.974)	6,674	1,250 (1.55E-04)	6,700	1,229 (0.637)
SETDB1	JASPAR Predicted	NA	NA	NA	NA	NA	NA
SETDB1	MotifMap Predicted	NA	NA	NA	NA	NA	NA
SETDB1	TRANSFAC Curated	NA	NA	NA	NA	NA	NA
SETDB1	TRANSFAC Predicted	NA	NA	NA	NA	NA	NA
SMAD4	CHEA	3,359	1,619 (2.74E-07)	3,368	765 (3.68E-19)	3,403	828 (5.01E-23)
SMAD4	ENCODE	NA	NA	NA	NA	NA	NA
SMAD4	JASPAR Predicted	NA	NA	NA	NA	NA	NA

TFs	Databases	Both sexes (6,284 genes)		Male (2,565 genes)		Female (2,706 genes)	
		# Target genes detected in rat frontal cortex	# Overlapping genes (*p* value)	# Target genes detected in rat frontal cortex	# Overlapping genes (*p* value)	# Target genes detected in rat frontal cortex	# Overlapping genes (*p* value)
SMAD4	MotifMap Predicted	8	3 (0.770)	8	2 (0.421)	8	3 (0.170)
SMAD4	TRANSFAC Curated	196	86 (0.589)	195	39 (0.200)	194	31 (0.839)
SMAD4	TRANSFAC Predicted	2,263	1,040 (0.058)	2,260	501 (3.01E-10)	2,280	518 (1.40E-08)
SOX5	CHEA	NA	NA	NA	NA	NA	NA
SOX5	ENCODE	NA	NA	NA	NA	NA	NA
SOX5	JASPAR Predicted	392	161 (0.921)	398	69 (0.552)	402	77 (0.378)
SOX5	MotifMap Predicted	10	3 (0.894)	10	2 (0.544)	11	2 (0.630)
SOX5	TRANSFAC Curated	219	104 (0.198)	214	56 (8.80E-04)	221	55 (1.01E-02)
SOX5	TRANSFAC Predicted	NA	NA	NA	NA	NA	NA
STAT1	CHEA	428	203 (0.111)	437	91 (3.78E-02)	430	89 (0.125)
STAT1	ENCODE	8,826	3,831 (0.999)	8,768	1,617 (9.92E-05)	8,816	1615 (0.711)
STAT1	JASPAR Predicted	1,178	551 (4.85E-02)	1,186	236 (1.33E-02)	1,188	222 (0.429)
STAT1	MotifMap Predicted	123	52 (0.716)	123	28 (0.080)	123	22 (0.602)
STAT1	TRANSFAC Curated	304	130 (0.741)	305	64 (0.063)	306	64 (0.148)
STAT1	TRANSFAC Predicted	31	14 (0.537)	32	5 (0.681)	32	7 (0.376)
TCF4	CHEA	3,026	1,389 (3.45E-02)	3,049	679 (1.31E-14)	3,063	726 (1.29E-16)
TCF4	ENCODE	NA	NA	NA	NA	NA	NA
TCF4	JASPAR Predicted	NA	NA	NA	NA	NA	NA
TCF4	MotifMap Predicted	358	176 (3.85E-02)	363	84 (3.28E-03)	368	87 (6.89E-03)
TCF4	TRANSFAC Curated	375	187 (1.83E-02)	379	96 (6.38E-05)	377	94 (9.40E-04)
TCF4	TRANSFAC Predicted	2,874	1,317 (4.79E-02)	2,898	541 (3.28E-02)	2,924	595 (1.90E-03)
TCF7L2	CHEA	526	257 (2.09E-02)	524	105 (0.067)	529	129 (3.23E-04)
TCF7L2	ENCODE	8,879	3,942 (0.546)	8,848	1,702 (2.17E-12)	8,871	1,752 (2.97E-07)
TCF7L2	JASPAR Predicted	NA	NA	NA	NA	NA	NA
TCF7L2	MotifMap Predicted	NA	NA	NA	NA	NA	NA
TCF7L2	TRANSFAC Curated	NA	NA	NA	NA	NA	NA
TCF7L2	TRANSFAC Predicted	NA	NA	NA	NA	NA	NA

TFs	Databases	Both sexes (6,284 genes)		Male (2,565 genes)		Female (2,706 genes)	
		# Target genes detected in rat frontal cortex	# Overlapping genes (*p* value)	# Target genes detected in rat frontal cortex	# Overlapping genes (*p* value)	# Target genes detected in rat frontal cortex	# Overlapping genes (*p* value)
THRA	CHEA	152	69 (0.436)	152	27 (0.497)	155	27 (0.663)
THRA	ENCODE	NA	NA	NA	NA	NA	NA
THRA	JASPAR Predicted	NA	NA	NA	NA	NA	NA
THRA	MotifMap Predicted	NA	NA	NA	NA	NA	NA
THRA	TRANSFAC Curated	NA	NA	NA	NA	NA	NA
THRA	TRANSFAC Predicted	187	85 (0.416)	191	43 (4.36E-02)	194	44 (0.079)
YY1	CHEA	1,954	807 (0.999)	1,944	363 (0.074)	1,953	334 (0.955)
YY1	ENCODE	11,132	4,983 (0.065)	11,125	2,146 (2.81E-26)	11,169	2,207 (6.64E-14)
YY1	JASPAR Predicted	3,648	1,631 (0.353)	3,676	709 (5.23E-04)	3,718	733 (1.22E-02)
YY1	MotifMap Predicted	7,898	3,660 (1.43E-07)	7,994	1,562 (3.32E-13)	8,017	1,676 (2.04E-17)
YY1	TRANSFAC Curated	633	259 (0.969)	645	145 (5.38E-04)	634	114 (0.641)
YY1	TRANSFAC Predicted	1,260	535 (0.934)	1,275	244 (0.057)	1,270	273 (2.27E-03)
ZBTB16	CHEA	NA	NA	NA	NA	NA	NA
ZBTB16	ENCODE	NA	NA	NA	NA	NA	NA
ZBTB16	JASPAR Predicted	NA	NA	NA	NA	NA	NA
ZBTB16	MotifMap Predicted	NA	NA	NA	NA	NA	NA
ZBTB16	TRANSFAC Curated	NA	NA	NA	NA	NA	NA
ZBTB16	TRANSFAC Predicted	1,091	480 (0.629)	1,087	199 (0.240)	1,102	217 (0.145)
