# Supplementary material for: Autism-Related Transcription Factors Underlying the Sex-Specific Effects of Prenatal Bisphenol A Exposure on Transcriptome-Interactome Profiles in the Offspring Prefrontal Cortex
Source: Int J Mol Sci. 2021 Dec 8;22(24):13201. doi: 10.3390/ijms222413201 (PMC8708761; doi:10.3390/ijms222413201)
Supplement: Supplementary file 1 [file ijms-22-13201-s001.zip › Table S15.pdf]

Table S15. List of primers for qRT-PCR analyses.

Gene	Forward primer (5′ > 3′)	Reverse primer (5′ > 3′)
Auts2	GTCCTCCAGGCCCTAGTCTC	CACACTGGGGCTATCCTTGT
Ankrd11	ATCAGGCAGTGCCATTCAGT	CATGCGGTCATACTTGTCGT
Ntng1	CTCAGACATAAAGGTGCGAGGA	TGGGGATGGGGAGGTATGAG
Dock4	TTTGATGTCCGGGAAGTGGC	TGTGGAGGTGATGCAGTACG
Syne1	ATTAGCCAGCGTGAGGAGTT	TCTGCTCGATCTTACTGTGGT
Rn18s	CTGGATACCGCAGCTAGGAA	GAATTTCACCTCTAGCGGCG
