# Supplementary material for: Autism-Related Transcription Factors Underlying the Sex-Specific Effects of Prenatal Bisphenol A Exposure on Transcriptome-Interactome Profiles in the Offspring Prefrontal Cortex
Source: Int J Mol Sci. 2021 Dec 8;22(24):13201. doi: 10.3390/ijms222413201 (PMC8708761; doi:10.3390/ijms222413201)
Supplement: Supplementary file 1 [file ijms-22-13201-s001.zip › Table S4.pdf]

Table S4: Neurological functions and disorders associated with DEGs in the offspring's prefrontal cortex in response to prenatal BPA exposure. The lists of differentially expressed genes in the prefrontal cortex of rat pups prenatally exposed to BPA when i) both male and female pups were combined into one group for each treatment and ii) each sex of pups was analyzed separately and uploaded to IPA software, and neurological functions and disorders associated with these BPA-responsive genes were predicted. Statistical significance was determined using Fisher's exact test and is shown as *p*-values. *P*-value < 0.05 is considered as significant.

**Neurological diseases/nervous system development and function associated with BPA responsive DEGs (*p*-value (#genes))**		
**Both sexes**	**Males**	**Females**
Cognitive impairment 8.32E-34 (565)	Mental retardation 1.97E-14 (123)	Dementia 1.07E-12 (188)
Neuromuscular disease 5.74E-23 (586)	Cognitive impairment 1.29E-13 (137)	Tauopathy 7.89E-12 (178)
Mental retardation 4.14E-21 (398)	Tauopathy 9.02E-12 (170)	Neuromuscular disease 1.53E-10 (218)
Tauopathy 1.15E-20 (373)	Dementia 4.27E-11 (174)	Dyskinesia 2.81E-10 (144)
Dementia 1.58E-20 (385)	Neuromuscular disease 7.53E-10 (205)	Cerebral degeneration 2.22E-09 (76)
Dyskinesia 5.58E-20 (417)	Dyskinesia 1.99E-08 (131)	Schizophrenia 3.49E-09 (132)
Epilepsy or neurodevelopmental disorder 3.57E-17 (401)	Proliferation of neuronal cells 3.40E-06 (27)	Ataxia 9.07E-06 (41)
Seizure disorder 4.16E-17 (354)	Parkinson's disease 5.61E-06 (68)	Growth of neurites 1.50E-05 (24)
Neuritogenesis 8.23E-28 (396)	Seizure disorder 1.59E-05 (90)	Proliferation of neuronal cells 3.14E-05 (26)
Proliferation of neuronal cells 3.60E-19 (319)	Epilepsy 1.76E-05 (80)	Cognitive impairment 3.51E-05 (112)

Neurological diseases/nervous system development and function associated with BPA responsive DEGs (*p*-value (#genes))		
**Both sexes**	**Males**	**Females**
Neurotransmission<br>2.38E-17 (250)	Growth of neurites<br>1.85E-05 (23)	Mental retardation<br>3.82E-05 (97)
