# Supplementary material for: Autism-Related Transcription Factors Underlying the Sex-Specific Effects of Prenatal Bisphenol A Exposure on Transcriptome-Interactome Profiles in the Offspring Prefrontal Cortex
Source: Int J Mol Sci. 2021 Dec 8;22(24):13201. doi: 10.3390/ijms222413201 (PMC8708761; doi:10.3390/ijms222413201)
Supplement: Supplementary file 1 [file ijms-22-13201-s001.zip › Table S5.pdf]

Table S5: Comparison analysis using BPA-responsive genes in both sexes, males, and females showing an association of BPA-responsive genes with ASD-related canonical pathways. Comparison analysis between BPA-responsive genes in both sexes, males only, and females only was performed by IPA software. The neurological-related canonical pathways were shown to be significantly affected in males but were not significantly affected in females, and vice versa. Statistical significance was determined using Fisher's exact test and is shown as *p*-values. *P*-value < 0.05 is considered as significant.

**Ingenuity canonical pathways**	***P*-values of DEGs in both sexes and each sex associated with neurological-related pathways (gene numbers)**		
	**Both sexes**	**Males**	**Females**
Axonal Guidance Signaling	**6.76E-07 (187)**	**1.00E-11 (105)**	**1.58E-11 (109)**
Synaptic Long-Term Depression	**5.25E-06 (80)**	**3.72E-05 (40)**	**1.15E-03 (37)**
NGF Signaling	**6.61E-06 (52)**	**6.46E-03 (25)**	**0.030 (23)**
Wnt/Ca+ pathway	**2.95E-05 (33)**	0.328 (9)	**0.022 (14)**
CREB Signaling in Neurons	**2.14E-04 (92)**	**1.48E-06 (51)**	**1.07E-03 (44)**
Androgen Signaling	**2.95E-04 (63)**	**3.09E-04 (31)**	**0.013 (27)**
GABA Receptor Signaling	**0.002 (41)**	**6.31E-04 (23)**	**5.10E-02 (18)**
Netrin Signaling	**0.010 (28)**	**9.00E-03 (15)**	0.120 (12)
Neuroinflammation Signaling Pathway	**0.015 (105)**	0.133 (43)	**0.021 (51)**
Estrogen Receptor Signaling	**0.020 (50)**	**0.030 (24)**	0.188 (21)
IL-6 Signaling	**0.040 (49)**	0.124 (21)	**0.019 (26)**
mTOR Signaling	**0.049 (70)**	**1.10E-02 (36)**	0.222 (30)
Neurotrophin/TRK Signaling	0.066 (31)	**9.00E-03 (18)**	0.103 (15)
Notch Signaling	0.370 (12)	0.465 (5)	**0.040 (9)**
