# Supplementary material for: Autism-Related Transcription Factors Underlying the Sex-Specific Effects of Prenatal Bisphenol A Exposure on Transcriptome-Interactome Profiles in the Offspring Prefrontal Cortex
Source: Int J Mol Sci. 2021 Dec 8;22(24):13201. doi: 10.3390/ijms222413201 (PMC8708761; doi:10.3390/ijms222413201)
Supplement: Supplementary file 1 [file ijms-22-13201-s001.zip › Table S9.pdf]

Table S9: Previously published autism-related transcriptome studies obtained from NCBI GEO DataSets and original articles used in the data-mining analysis of BPA-responsive genes.

Studies	Author and Year	Sample region	Sample size
Immune transcriptome alterations in the temporal cortex of subjects with autism (*p*-value < 0.05)*	Garbett et al., 2008	Temporal cortex (superior temporal gyrus)	6 ASD and 6 CTRL
Transcriptomic analysis of autistic brain reveals convergent molecular pathology (GSE28521)	Voineagu et al., 2011	Frontal cortex (BA9)	16 ASD and 16 CTRL
		Temporal cortex (BA41/42 or BA22), BA44/45	13 ASD and 13 CTRL
		Cerebellum (vermis)	10 ASD and 11 CTRL
Age-Dependent Brain Gene Expression and Copy Number Anomalies in Autism Suggest Distinct Pathological Processes at Young Versus Mature Ages (GSE28475)	Chow et al., 2012	Prefrontal cortex (BA9/46)	32 ASD and 33 CTRL
Brain transcriptional and epigenetic associations with the autistic phenotype (GSE38322)	Ginsberg et al., 2012	Cerebellum	8 ASD and 8 CTRL
		Occipital (BA19)	6 ASD and 4 CTRL
Genome-wide changes in lncRNA, splicing, and regional gene expression patterns in autism (FDR adj. *p*-value <0.05)*	Parikshak et al., 2015	Frontal (BA9) and temporal cortex (BA41-42-22)	26 ASD and 33 CTRL

* DEG lists obtained from original article
