# Supplementary figures and images for: Autism-Related Transcription Factors Underlying the Sex-Specific Effects of Prenatal Bisphenol A Exposure on Transcriptome-Interactome Profiles in the Offspring Prefrontal Cortex
Source: Int J Mol Sci. 2021 Dec 8;22(24):13201. doi: 10.3390/ijms222413201 (PMC8708761; doi:10.3390/ijms222413201)

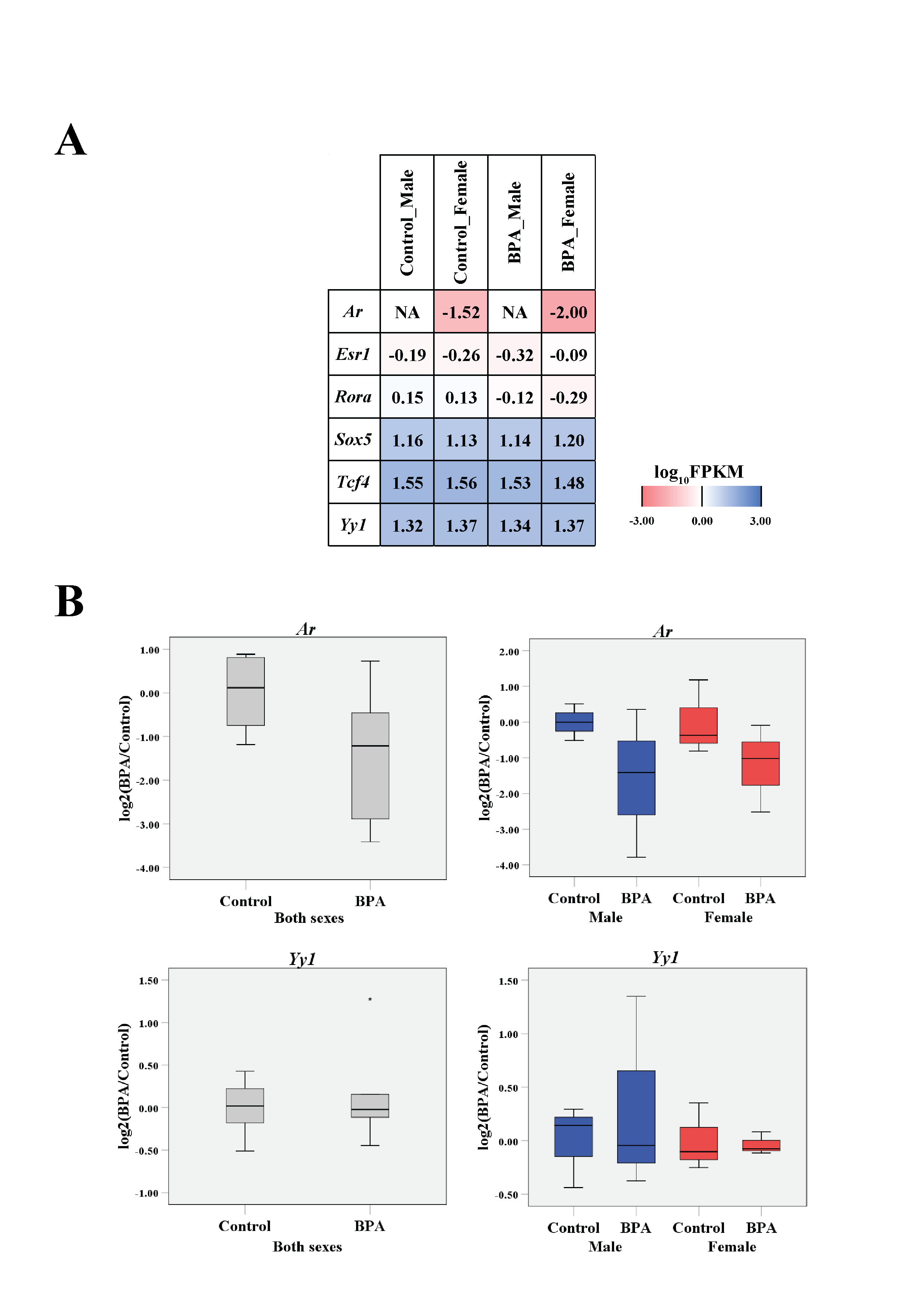

Supplement: Supplementary file 1 [file ijms-22-13201-s001.zip › Figure S3.tif]
